# Epstein-Barr Virus Early Protein BFRF1 Suppresses IFN-β Activity by Inhibiting the Activation of IRF3

**DOI:** 10.3389/fimmu.2020.513383

**Published:** 2020-12-17

**Authors:** Ping Wang, Yangxi Deng, Yingjie Guo, Zuo Xu, Yiwen Li, Xiaowen Ou, Li Xie, Manjiao Lu, Jiayi Zhong, Bolin Li, Li Hu, Shenyu Deng, Tao Peng, Mingsheng Cai, Meili Li

**Affiliations:** ^1^The Second Affiliated Hospital, State Key Laboratory of Respiratory Disease, Guangdong Provincial Key Laboratory of Allergy & Clinical Immunology, Sino-French Hoffmann Institute, School of Basic Medical Science, Guangzhou Medical University, Guangzhou, China; ^2^Centralab, Shenzhen Center for Chronic Disease Control, Shenzhen, China; ^3^State Key Laboratory of Respiratory Diseases, Sino-French Hoffmann Institute, Guangzhou Medical University, Guangzhou, China; ^4^South China Vaccine Corporation Limited, Guangzhou, China

**Keywords:** innate immunity, EBV BFRF1, IRF3, IFN-β, ISG54

## Abstract

Epstein-Barr virus (EBV) is the causative agent of infectious mononucleosis that is closely associated with several human malignant diseases, while type I interferon (IFN-I) plays an important role against EBV infection. As we all know, EBV can encode some proteins to inhibit the production of IFN-I, but it’s not clear whether other proteins also take part in this progress. EBV early lytic protein BFRF1 is shown to be involved in viral maturation, however, whether BFRF1 participates in the host innate immune response is still not well known. In this study, we found BFRF1 could down-regulate sendai virus-induced IFN-β promoter activity and mRNA expression of IFN-β and ISG54 during BFRF1 plasmid transfection and EBV lytic infection, but BFRF1 could not affect the promoter activity of NF-κB or IRF7. Specifically, BFRF1 could co-localize and interact with IKKi. Although BFRF1 did not interfere the interaction between IKKi and IRF3, it could block the kinase activity of IKKi, which finally inhibited the phosphorylation, dimerization, and nuclear translocation of IRF3. Taken together, BFRF1 may play a critical role in disrupting the host innate immunity by suppressing IFN-β activity during EBV lytic cycle.

## Introduction

Innate immunity is the first line of host conservative and rapid defense against pathogen invasion, of which type I interferon (IFN-I) plays an important role in the antiviral immune response (1). Along with viral infection, the pattern recognition receptors such as toll-like receptors (TLRs) (2), retinoic acid-inducible gene (RIG)-I-like receptors (RLRs), and nucleotide-binding oligomerization domain (NOD)-like receptors (NLRs) (3, 4), can recognize viral pathogen-associated molecular patterns (PAMPs), including genomic DNA, double-stranded RNA (dsRNA) with 5′-triphosphate end, single-stranded RNA (ssRNA), and viral proteins. RNA helicases retinoic acid inducible gene 1 (RIG-I) and melanoma differentiation-associated gene 5 (MDA-5) are the most vital RLRs and are reported to exert essential roles in the detection of intracellular dsRNA, which signal through IFN promoter stimulator 1 (IPS-1) to activate the kinases TANK-binding kinase 1 (TBK1) and inducible IκB kinase (IKKi; also known as inhibitor of κB kinase ϵ, IKKϵ), to finally phosphorylate IFN regulatory factor 3 (IRF3), this promotes the nuclear translocation of IRF3 and subsequent induction of IFN-I, proinflammatory cytokines and series interferon-stimulated genes (ISGs) (5–8).

Epstein-Barr virus (EBV), also called human herpesvirus 4 (HHV-4), is associated with the development of a wide spectrum of B-cell lymph proliferative disorders, such as Burkitt’s lymphoma (BL), post transplant lymph proliferative disorder (PTLD), Hodgkin and non-Hodgkin lymphomas, as well as epithelial cancers (including nasopharyngeal carcinoma [NPC] and some forms of gastric carcinoma) (9). It’s shown that many EBV-encoded gene products are involved in the innate immunity, and some of which can stimulate the production of IFN-I. EBV-encoded small nuclear RNA 1 (EBER1) and EBER2 act as ligand of RIG-I to activate IRF3 (10, 11). The C-terminal activation region (CTAR) of LMP1 also can activate NF-κB and IRF7 upon superinfection (12). Moreover, EBNA2 can stimulate IFN-β expression and ISGF3 activity in BL cell lines (13). However, some EBV-encoded gene products are demonstrated to inhibit the production of IFN-β. BGLF4 interplays with IRF3 to abolish its activity in reactivated EBV-positive cells (14). BZLF1 interacts with IRF7 to inhibit its activity (15). BRLF1 reduces the expression of IRF3 and IRF7, thereby inhibiting IFN-β and promoting viral replication (16). LMP2A and LMP2B abrogate IFN-β signaling cascade by promoting the circulation of I and II IFN receptors IFNAR and IFNGR (17). Furthermore, EBV-encoded RNA miR-BART6-3p also inhibits EBV-triggered IFN-β response and facilitates EBV infection through targeting the 3′UTR of RIG-I mRNA (18).

The herpesviral UL34 family contains herpes simplex virus 1 (HSV-1) UL34, varicella-zoster virus (VZV) ORF24, murine cytomegalovirus (MCMV) M50, Kaposi’s sarcoma-associated herpesvirus (KSHV) p29 and EBV BFRF1, etc. Previous studies have shown that the nuclear membrane targeted type II membrane protein HSV-1 UL34 can interact with UL31 to form the heterodimer-nuclear egress complex (NEC), and absence of any one of them will prevent the nuclear egress of viral nucleocapsid (19, 20) and primary envelope (21–23). MCMV M50 and the M53 (homologous to HSV-1 UL31) also form a complex to help the virus nuclear export (24). Besides, KSHV p29 interplays with p33 (homologous to HSV-1 UL31) to co-localize at the nuclear membrane, and p29 is responsible for the hyperphosphorylation and delocalization protein of emerin, which is essential for the maturation of viral nucleocapsid (25).

BFRF1 is an EBV-encoded early lytic protein (26), which can regulate multiple viral and cellular functions, including viral maturation, BFLF2 nuclear membrane targeting, lamin B1 binding, recruitment of ESCRT machinery, and cytoplasmic vesicles formation (27–30). BFRF1 and BFLF2 together can form NEC, which is involved in the early step of EBV nuclear egress. Nevertheless, it’s a wonder whether BFRF1 also plays a regulatory role in the host innate immunity. In the present study, we found that BFRF1 inhibited the IFN-β production through its interaction with IKKi to restrain the kinase activity of IKKi and suppressing the activation of IRF3 during BFRF1 plasmid transfection and EBV lytic infection.

## Materials and Methods

### Virus and Cells

Sendai virus (SeV) was propagated in chicken embryo and titered in our lab. HeLa, COS-7, and human embryonic kidney (HEK) 293T cells were grown in Dulbecco’s modified MEM (DMEM, Gibco-BRL) supplemented with 10% heat inactivated fetal bovine serum (FBS, Gibco-BRL) at the temperature of 37°C in a humidified incubator with 5% CO_2_. Hone1-EBV cells (kindly provided by Prof. Sai Wah Tsao, University of Hong Kong, Hong Kong, China) are EBV positive nasopharyngeal carcinoma cell lines that can be reactivated by a sequence-specific DNA-binding protein, BZLF1 [also called Z, Zebra or EB1, encoded by immediate-early *BZLF1* gene (31, 32)].

### Antibodies

Mouse anti-Flag (DYKDDDDK), anti-Myc, and anti-hemagglutinin (HA) monoclonal antibodies (mAbs) were obtained from ABmart. Rabbit anti-Flag mAb and anti-IRF3 polyclonal antibody (pAb) were purchased from Proteintech. Rabbit anti-IKKi and anti-β-actin pAbs were provided by ABclonal. Cy5-conjugated goat anti-rabbit IgG, ﬂuorescein isothiocyanate (FITC)-conjugated goat anti-mouse IgG, and RBITC-conjugated goat anti-rabbit IgG were bought from BBI Life Sciences. Rabbit phospho-IRF3 (ser396) mAb, alkaline phosphatase (AP) conjugated goat anti-mouse IgG, and goat anti-rabbit IgG were obtained from Cell Signaling Technology.

### Plasmids Construction

To construct Flag-tagged BFRF1 expression plasmid, the open reading frame of *BFRF1* was polymerase chain reaction (PCR) amplified from the bacterial artificial chromosome (BAC) DNA of B95-8 strain of EBV (174-kb BAC) (33), with forward primer 5′-TTA AGC TTC CGA ATT CAT GGC GAG CCC GGA AGA GAG and reverse primer 5′-TTG CGG CCG CAG GAT CCA AGG TCC ACC TCA GAA ACA TCA G. Then, the purified PCR product was digested with *EcoR*I and *BamH*I and inserted into the corresponding digested Flag vector (regenerated from pFlag-CMV-2, Sigma) to yield pBFRF1-Flag, as described previously (34–37). pBFRF1-Myc, pIKKi-HA, pBZLF1-HA (Zta-HA), and pBGLF4-HA were also constructed with similar methods, using pMyc-N1 or pHA-N1 vector (regenerated from pEYFP-N1, Clontech). Besides, one pair of oligonucleotide sequences 5′-GGG TCT CTC AAC GGA TGT TGA and 5′-CTC AAC TCA CGT GTC TAG TGT C (38–44) was inserted into the good RNAi product of Oligoengine pSuper-retro-puro (Oligoengine) that can effectively remove off-target of the target gene, to construct RNA interference expression plasmids pSuper-shBFRF1-retro-puro and pSuper-shRandom-retro-puro [a good off-target control (45–48)], respectively. Other gift plasmids were provided by Drs. John Hiscott (IFN-β-Luc) (49), Rongtuan Lin (ISRE-Luc and pIKKi-Flag) (50), Stephan Ludwig (IRF3-Luc) (51), M. Pitha (IRF7-Luc) (52), Takashi Fujita (pRIG-IN-Flag) (53), Takemasa Sakaguchi (pIRF3-HA) (54), Yi-Ling Lin (pIRF3-Flag, pIRF3/5D-Flag, and pIRF7/6D-Flag) (55), Zhengli Shi (pRL-TK and NF-κB-Luc), Chunfu Zheng (pTBK1-Myc and pRIG-I-Flag), and Jun Cui (pMAVS-Flag, pIKKi-Myc, pTRAF3-Flag, and pTBK1-Flag).

### Indirect Immunofluorescent Assays (IFA)

The IFA was carried out as described previously (41, 56–60). In brief, a 14 mm circle microscope cover glass (NEST) was placed in 24-well plate (Corning), then COS-7 or HeLa cells were seeded overnight to 80% confluence and transfected with the indicated plasmids by polyethylenimine (PEI) according to the manufacturer’s instructions. At 24 h post-transfection, cells were mock-treated or treated with SeV (100 hemagglutination units [HAU]/ml) for 16 h, then cells were washed three times with PBS and fixed with 4% paraformaldehyde (Beyotime Biotechnology) for 30 min at 37°C, and incubated in 0.1% Triton X-100 (Beyotime Biotechnology) for 10 min. After that, the cells were washed three times with PBS and blocked with 5% BSA for 1 h at 37°C, followed by incubation with primary Abs (anti-Flag, anti-HA, anti-IRF3, or anti-Myc) for 12 h at 4°C, subsequently cells were washed three times with TBST, and incubated with second Ab FITC-conjugated goat anti-mouse IgG, RBITC-conjugated goat anti-rabbit IgG, or Cy5-conjugated goat anti-rabbit IgG for 1 h. Finally, cells were counterstained with Hoechst to visualize the nuclear DNA for 5 to 10 min. The microscope cover glass place microscope slides (biosharp) were obtained with anti-fluorescence quenching reagent (Biosharp) and then fixed with nail polish, and images were eventually captured with 630× of confocal microscope (Leica SP8, 81-933). All scale bars indicate 10 μm.

### Dual-Luciferase Reporter (DLR) Assays

The DLR assays were performed as described previously (61). HEK293T cells were seeded on 24-well plate at a density of 1 × 10^5^ cells per well overnight, then cells were co-transfected with 100 ng of promoter reporter expression plasmid, 10 ng of pRL-TK control plasmid and the indicated amounts of expression plasmid. At 24 h post-transfection, cells were mock-infected or infected with 100 HAU/ml SeV for 16 h, followed by washing two times with PBS. Then, cell lysates were collected and luciferase activity was assessed using a luciferase assay kit (Promega). Finally, the data for DLR were detected by Glomax Discover. Data were normalized for transfection efficiency by detecting renilla luciferase activity and firefly luciferase activity, and results were demonstrated as the ratio between firefly and Renilla luciferase. Data were presented as means ± standard deviations (SD) from three independent experiments.

### RNA Isolation and Real-Time Quantitative PCR (RT-qPCR)

HEK293T cells seeded on six-well plate (Corning) overnight to 80% confluence were transfected with the indicated plasmids, or Hone1-EBV cells were transfected with the BZLF1 expression plasmid to induce the lytic infection of EBV. After plasmid transfection or EBV induction, cells were mock-treated or treated with 100 HAU/ml SeV for 16 h, then total RNA was extracted with TRIzol reagent (Invitrogen) for reverse transcription to cDNA with RT reagent (YEASEN). The acquired cDNA was used as a template for RT-qPCR, to detect the mRNA expression of glyceraldehyde-3-phosphate dehydrogenase (GAPDH), human IFN-α, IFN-β, and ISG54, with qPCR reagent (YEASEN) using qPCR instrument (BIO-RAD, CFX96). Primers used for GAPDH (forward primer 5′-AGG TCG GTG TGA ACG GAT TTG and reverse primer 5′-TGT AGA CCA TGT AGT TGA GGT CA), IFN-β (forward primer 5′-ATG ACC AAC AAG TGT CTC CTC C and reverse primer 5′-GGA ATC CAA GCA AGT TGT AGC TC), and ISG54 (forward primer 5′-GGA GGG AGA AAA CTC CTT GGA and reverse primer 5′-GGC CAG TAG GTT GCA CAT TGT) were referred to the study of Bing Tian (62). Primers 5′-CAG AGT CAC CCA TCT CAG C (forward primer) and 5′-ATT TGT GCC AGG AGC ATC (reverse primer) were designed to detect the mRNA of IFN-α.

### Co-Immunoprecipitation Assays and Western Blot Analysis

The co-immunoprecipitation (Co-IP) and western blot (WB) assays were carried out as described previously (59, 60, 63–69). In brief, HEK293T cells were seeded on 10 cm petri-dish (Corning) and incubated to ~80% confluence, then cells were co-transfected with 20 μg of plasmid combinations tagged with Myc, Flag, or HA. At 24 h post-transfection, cells were harvested and lysed on ice with 800–1,000 μl RIPA lysis buffer (Beyotime Biotechnology) for 30 min. After that, cell lysates were divided into two parts, 10% lysates were directly prepared as the lysates sample, and 90% lysates were incubated with the indicated Ab (anti-Flag or anti-HA) or nonspecific control mouse antibody (IgG) at 4°C for 6 to 12 h, then the antibody-containing lysates were incubated with 50 μl slurry of protein A/G PLUS-Agarose (Santa Cruz) at 4°C overnight. The bead complex was washed at least three times with 1 ml of PBS. Finally, cell lysates and bead protein complex were subjected to WB analysis to detect the potential interaction of virus-host proteins. The original WB results were shown in the section of **Supplementary Material**.

### Native PAGE

Native PAGE was carried out as described previously (70). HEK293T cells were seeded on six-well plate overnight to 70% confluence, then cells were transfected with the indicated plasmids. At 24 h post-transfection, cells were mock-treated or treated with 100 HAU/ml SeV for 8 or 16 h, subsequently cells were harvested and lysed with weak RIPA lysis buffer (Beyotime Biotechnology) at 4°C for 30 min. Gels were pre-run with 25 mM Tris and 192 mM glycine (pH 8.4) with 2% deoxycholate (DOC) in a cathode chamber for 30 min at 75 V. Samples in native sample buffer (1 M Tris-Hcl [pH 6.8], 15% glycerol, and 2% bromophenol blue) were then size fractionated by electrophoresis for 120 min at 75 V and transferred to nitrocellulose membrane (BBI Life Sciences) for WB analysis. For analyzing the BFRF1 inhibitory effect of IRF3 dimerization during EBV lytic infection, Hone1-EBV cells were co-transfected with the BZLF1 expression plasmid and pSuper-shBFRF1-retro-puro expression plasmid or pSuper-shRandom-retro-puro expression plasmid, then other experimental procedures were performed as described above.

### Statistical Analysis

All data were analyzed with Student’s T tests in GraphPad Prism 5 software. Here, ns indicates not significant; * indicates *P* value <0.05; ** indicates *P* value <0.01; *** indicates *P* value <0.001; and **** indicates *P* value <0.0001. A *P* value <0.05 was considered significant.

## Results

### BFRF1 Suppresses SeV-Mediated IFN-β Transcriptional Activation

**Figure 1:**
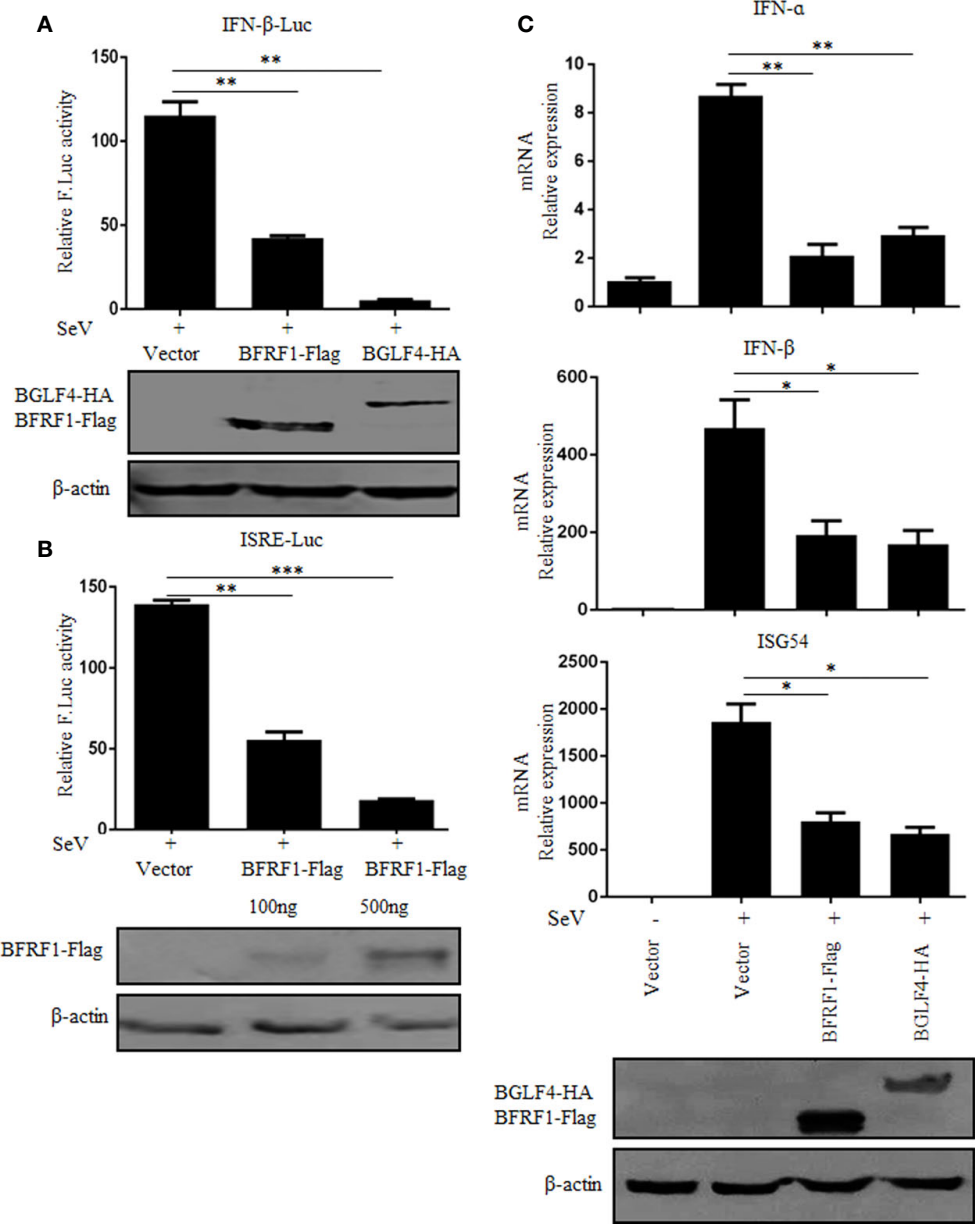
BFRF1 suppresses SeV-mediated activation of IFN-β and ISRE promoters. Vector, BFRF1-Flag, or BGLF4-HA expression plasmid was co-transfected with pRL-TK control plasmid and IFN-β-Luc **(A)** or ISRE-Luc **(B)** reporter plasmid into HEK293T cells. At 24 h post-transfection, cells were infected with 100 HAU/ml of SeV for 16 h. Cell lysates were then collected, and luciferase activity was measured by DLR. The expression of BFRF1 or BGLF4 protein was also detected by WB using anti-Flag or anti-HA mAb, and β-actin was used to verify equal loading of protein in each lane. **(C)** Expression plasmid BFRF1-Flag, BGLF4-HA or vector was transfected into HEK293T cells, at 24 h post-transfection, cells were mock-infected or infected with 100 HAU/ml SeV for 16 h. Cells were then lysed and RNA was extracted for RT-qPCR analysis for IFN-α,IFN-β and ISG54. The expression of BFRF1 or BGLF4 protein was also detected by WB using anti-Flag or anti-HA mAb, and β-actin was used to verify equal loading of protein in each lane. Statistical analysis was performed using student’s t test. ns, not significant; *P < 0.05; **P < 0.01; ***P < 0.001.

IFN-α/β play an essential role in antiviral innate immunity (71, 72). Here, to investigate whether BFRF1 can regulate IFN-β transcriptional activity, expression plasmid BFRF1-Flag, BGLF4-HA, or vector was co-transfected with reporter genes IFN-β-Luc and pRL-TK into HEK293T cells. At 24 h post-transfection, cells were treated with 100 HAU/ml SeV for 16 h, and DLR assays were performed. As a result, the activity of IFN-β promoter was obviously induced by SeV, which was significantly inhibited by the ectopic expression of BFRF1 (**Figure 1A**), with similar inhibitory effect to the positive control BGLF4 (14). Furthermore, to explore whether BFRF1 regulate IFN-induced gene expression, the activation of interferon-stimulated response element (ISRE) promoter was detected by DLR assays. As shown in **Figure 1B**, the ISRE promoter activity was also inhibited by BFRF1. To validate these results, expression plasmid BFRF1-Flag, BGLF4-HA, or vector was transfected into HEK293T cells to see whether BFRF1 can regulate the mRNA expression of IFN-β and its downstream gene, such as ISG54. The results showed BFRF1 could reduce SeV-induced mRNA expressions of IFN-β and ISG54 when compared to BGLF4 (**Figure 1C**), confirming BFRF1 could suppress SeV-mediated IFN-β transcriptional activation. Besides, BFRF1 also could restrain SeV-mediated IFN-α transcriptional activation (**Figure 1C**).

### Knockdown of BFRF1 Enhances IFN-β Transcriptional Activity During EBV Infection

**Figure 2:**
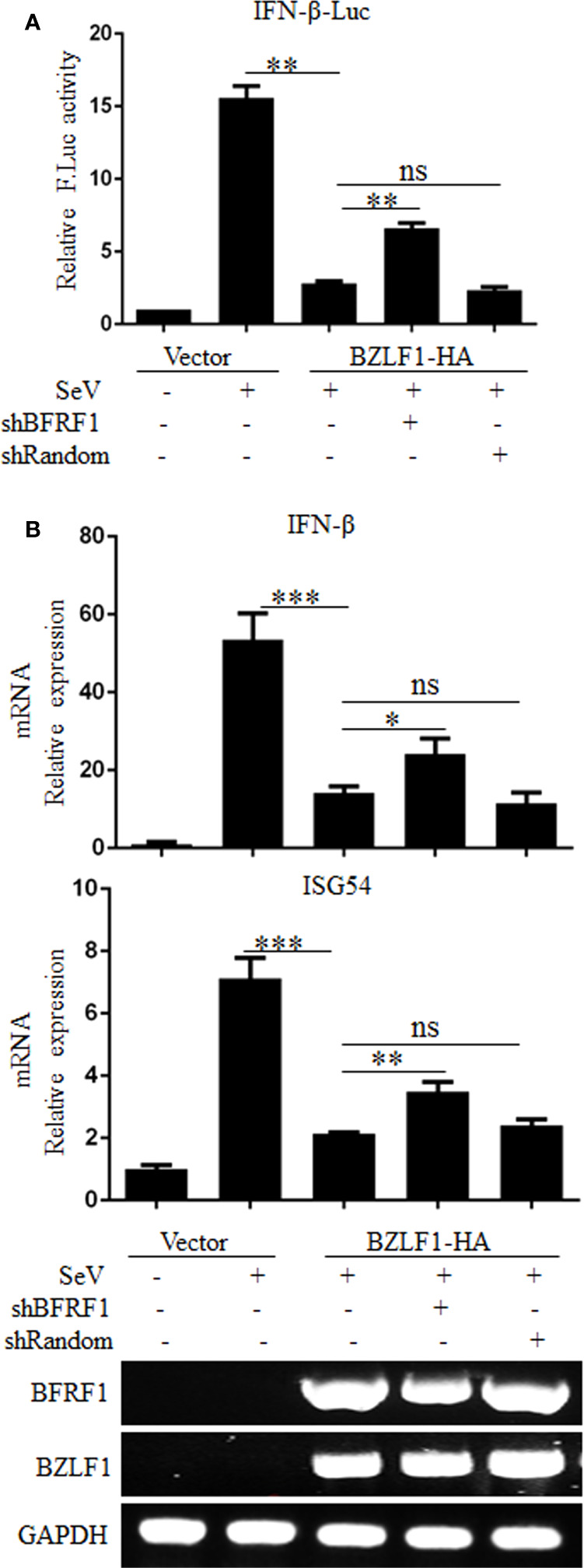
Knockdown of BFRF1 during EBV lytic infection increases the transcriptional activations of IFN-β and ISG. **(A)** Hone1-EBV cells were co-transfected with reporter IFN-β-Luc, BZLF1-HA expression plasmid and pSuper-shBFRF1-retro-puro or pSuper-shRandom-retro-puro expression plasmid. At 24 h post-transfection, cells were mock-infected or infected with 100 HAU/ml SeV for 16 h. Cell lysates were then collected, and luciferase activity was measured by DLR. **(B)** Hone1-EBV cells were co-transfected with BZLF1-HA expression plasmid and pSuper-shBFRF1-retro-puro or pSuper-shRandom-retro-puro expression plasmid. At 24 h post-transfection, cells were mock-infected or infected with 100 HAU/ml SeV for 16 h. Cells were then lysed and RNA was extracted for reverse transcription into cDNA and qPCR analysis for the mRNA levels of IFN-β and ISG54, and RT-PCR analysis for the mRNA levels of BFRF1, BZLF1, and GAPDH. Statistical analysis was performed using Student’s t test. ns, not significant; *P < 0.05; **P < 0.01; ***P < 0.001.

To further confirm the physiological function of BFRF1, the expression of BFRF1 was firstly knocked down in EBV-positive Hone1 cells co-transfected with reporter IFN-β-Luc, BZLF1-HA, and pSuper-shBFRF1-retro-puro expression plasmid or pSuper-shRandom-retro-puro expression plasmid, and DLR assays showed that the SeV-induced IFN-β promoter activity was inhibited after EBV was reactivated by BZLF1, but this inhibition is weakened when knockdown the expression of BFRF1 in EBV-positive Hone1 cells (**Figure 2A**). To further verify this result, RT-qPCR was carried out and showed that the mRNA expressions of IFN-β and ISG54 were reduced after EBV was reactivated, while these mRNA expressions were up-regulated when BFRF1 was knocked down in EBV-positive Hone1 cells (**Figure 2B**). These data indicated that BFRF1 could down-regulate IFN-β and downstream ISG54 during EBV infection.

### BFRF1 Restrains the Promoter Activation of IRF3, but Not IRF7 or NF-κB

**Figure 3:**
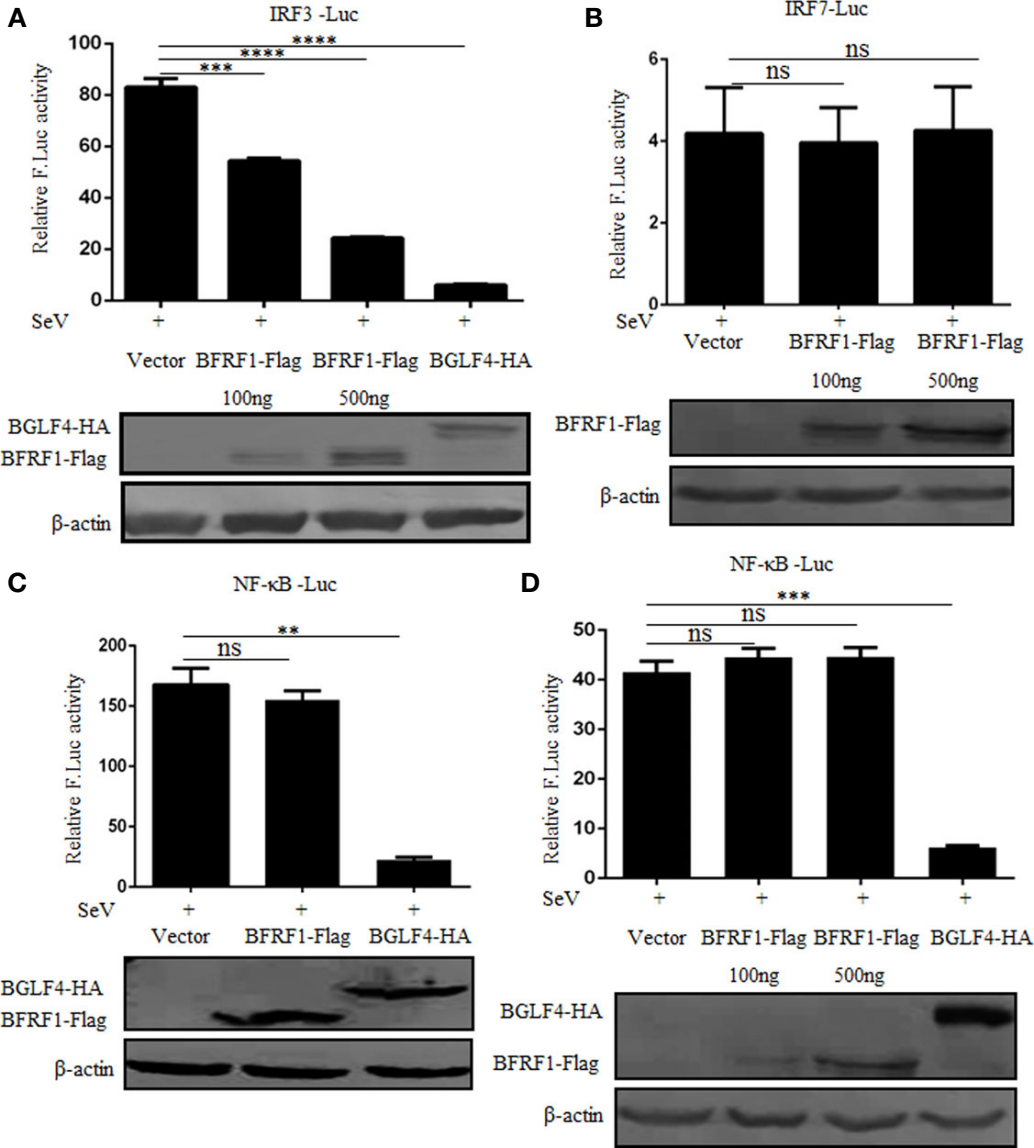
BFRF1 restrains the activation of IRF3 promoter, but not IRF7 or NF-κB promoter. Vector, BFRF1-Flag (100 or 500 ng) or BGLF4-HA (500 ng) expression plasmid was co-transfected with pRL-TK control plasmid and IRF3-Luc **(A)**, IRF7-Luc **(B)**, or NF-κB-Luc **(C, D)** reporter plasmid into HEK293T cells. At 24 h post-transfection, cells were infected with 100 HAU/ml SeV for 16 h. Cell lysates were then collected, and luciferase activity was measured by DLR. The expression of BFRF1 or BGLF4 protein was also detected by WB using anti-Flag or anti-HA mAb, and β-actin was used to verify equal loading of protein in each lane. Statistical analysis was performed using student’s t test. ns, not significant; **P < 0.01; ***P < 0.001; ****P < 0.0001.

The transcription activation of IFN-β depends on the synergistic interactions among IRFs, NF-κB, and other transcription factors that bind to distinct regulatory domains of the IFN-β promoter (73). Here, to explore whether the activation of NF-κB and IRFs were inhibited by BFRF1, different concentrations of BFRF1-Flag expression plasmid or vector was co-transfected with reporter genes pRL-TK and NF-κB-Luc, IRF3-Luc or IRF7-Luc into HEK293T cells. At 24 h post-transfection, cells were treated with 100 HAU/ml SeV for 16 h, and DLR assays were performed. As shown in **Figure 3**, the promoter activities of IRF3, IRF7 and NF-κB were obviously induced by SeV infection. However, ectopic expression of BFRF1 could dose-dependent inhibit SeV-mediated IRF3 promoter activity (**Figure 3A**), but not IRF7 (**Figure 3B**) or NF-κB (**Figures 3C, D**) promoter activity. Accordingly, these results demonstrated that BFRF1 significantly inhibited the transcriptional activation of IFN-β through IRF3, but not IRF7 or NF-κB.

### BFRF1 Represses IFN-β Promoter Activity at the Level of IKKi

**Figure 4:**
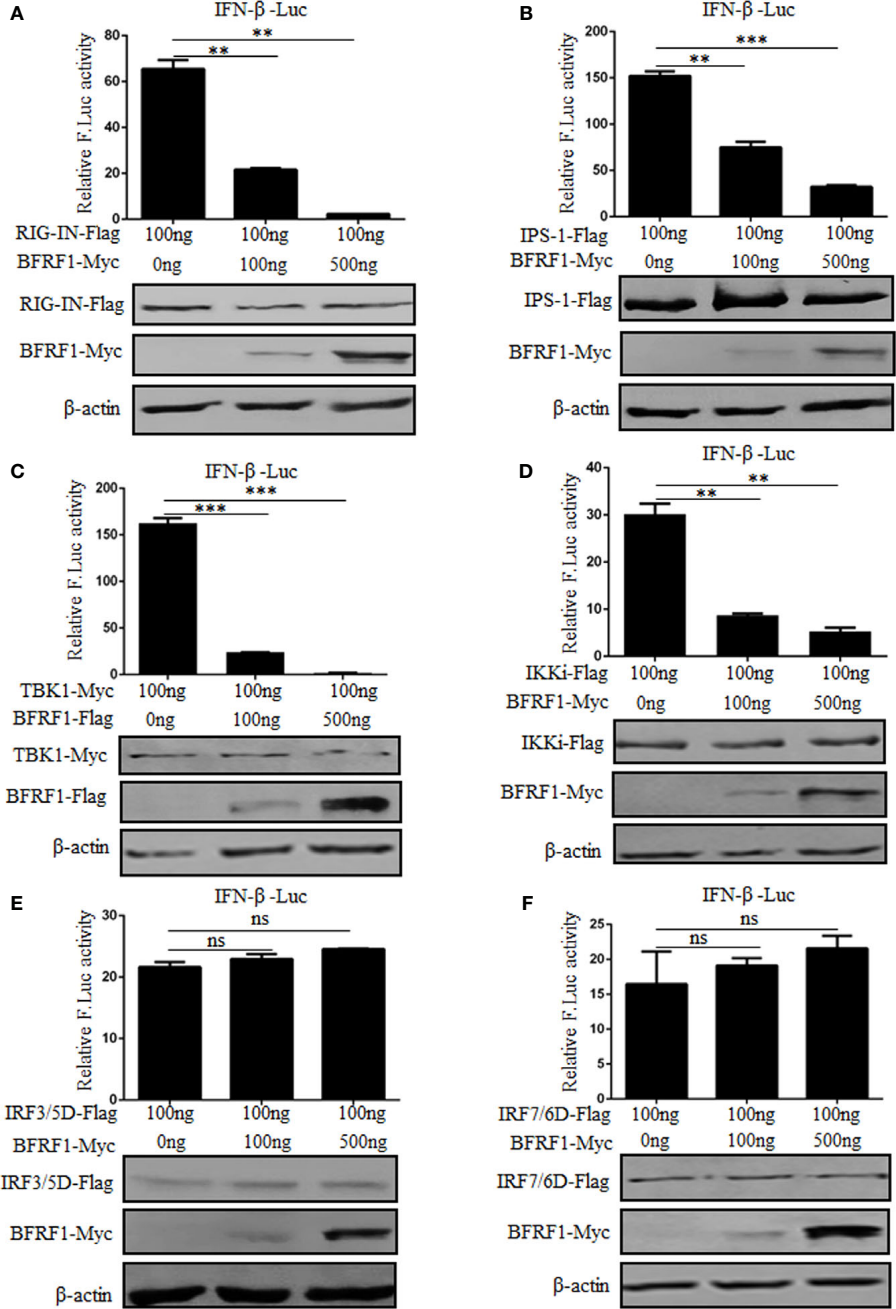
BFRF1 dose-dependent represses IFN-β promoter activity at the level of IKKi. IFN-β-Luc reporter and pRL-TK control plasmid were co-transfected with RIG-IN **(A)**, IPS-1 **(B)**, TBK1 **(C)**, IKKi **(D)**, IRF3/5D **(E)**, or IRF7/6D **(F)** expression plasmid into HEK293T cells, together with the indicated amounts of BFRF1 expression plasmid for 24 h, then luciferase activity was analyzed by DLR. The expressions of related adaptor and viral proteins were also detected by WB using specific tag Abs, and β-actin was used to verify equal loading of protein in each lane. Statistical analysis was performed using Student’s t test. ns, not significant; **P < 0.01; ***P < 0.001.

In order to examine at which level BFRF1 inhibits the production of IFN-β, different stimuli were used in HEK293T cells to induce the IFN-β reporter activity. RIG-IN (a constitutively active variant containing only the amino-terminal CARD of RIG-I), IPS-1, TBK1, IKKi, IRF-3/5D (a phosphorylated form of IRF-3), or IRF-7/6D (a phosphorylated form of IRF-7) was overexpressed to analyze the IFN-β reporter activity in the presence of various concentrations of BFRF1 expression plasmid. As results, overexpression of signaling components RIG-IN, IPS-1, TBK1, and IKKi activated the IFN-β promoter, which was significantly inhibited by BFRF1 in a dose-dependent manner (**Figures 4A–D**). However, IRF-3/5D and IRF-7/6D induced IFN-β promoter activity were not affected by BFRF1 (**Figures 4E, F**). Collectively, these results suggested that BFRF1 likely acted at the level of IKKi to inhibit the production of IFN-β.

### BFRF1 Co-Localizes and Interacts With IKKi

**Figure 5:**
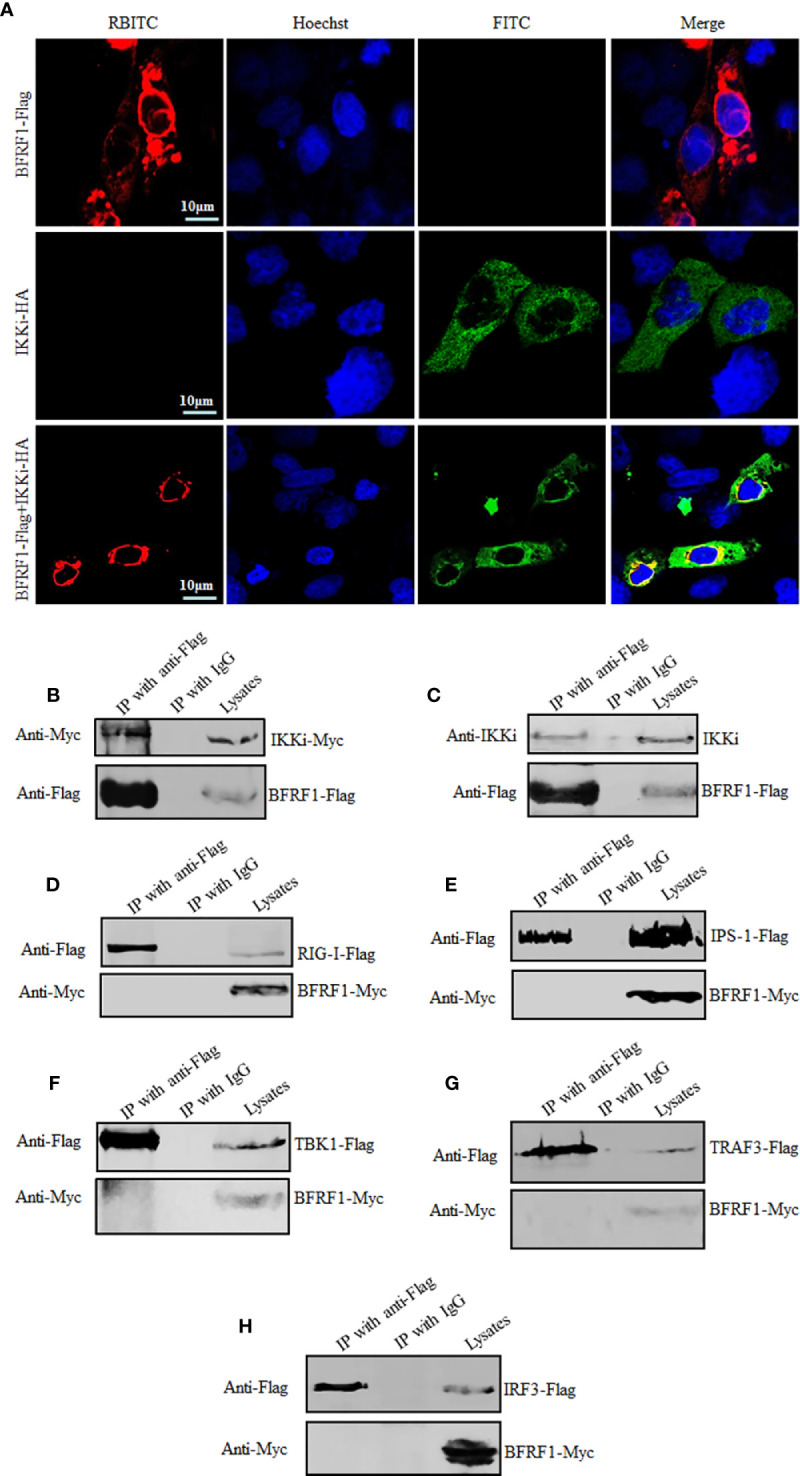
BFRF1 co-localizes and interacts with IKKi. **(A)** COS-7 cells were transfected with expression plasmid pIKKi-HA or BFRF1-Flag, or co-transfected with plasmids combination pIKKi-HA and BFRF1-Flag. At 24 h post-transfection, IFA analysis was performed with primary Abs anti-HA and anti-Flag mAb, and their corresponding fluorescent secondary Abs FITC-conjugated goat anti-mouse IgG (green) and RBITC-conjugated goat anti-rabbit IgG (red), respectively. Cells were counterstained with Hoechst to visualize the nuclear DNA (blue) for 5 to 10 min. Images were obtained by confocal microscopy using a 63× lens objective. All scale bars indicate 10 μm. **(B**, **C)** HEK293T cells were transfected with plasmid BFRF1-Flag **(C)** or co-transfected with plasmids combination BFRF1-Flag and pIKKi-Myc **(B)**. At 24 h post-transfection, cells were lysed and immunoprecipitated with anti-Flag mAb or mouse nonspecific IgG, then WB analysis was performed using the indicated Abs. **(D–H)** HEK293T cells were co-transfected with plasmids combination of pRIG-I-Flag/pBFRF1-Myc **(D)**, pIPS-1-Flag/pBFRF1-Myc **(E)**, pTBK1-Flag/pBFRF1-Myc **(F)**, pTRAF3-Flag/pBFRF1-Myc **(G)**, or pIRF3-Flag/pBFRF1-Myc **(H)**. At 24 h post-transfection, cells were lysed and immunoprecipitated with anti-Flag mAb or mouse nonspecific IgG, then WB analysis was performed using the indicated Abs.

In order to probe the inhibition mechanism of IFN-β transcriptional activation by BFRF1, IFA and Co-IP assays were carried out to determine whether BFRF1 could co-localize and interact with IKKi. COS-7 cells, with the cytoplasm and nucleus relatively larger than that of HEK293T cells for observing the subcellular localizations of specific proteins in different cell compartments, were transfected with the expression plasmid pIKKi-HA or pBFRF1-Flag, or co-transfected with plasmids combination pIKKi-HA and pBFRF1-Flag, then IFA was performed and showed that BFRF1 and IKKi co-localized at the perinuclear region (**Figure 5A**). Besides, HEK293T cells, a common cell model for analyzing the protein-protein interaction, were transfected with the expression plasmid pBFRF1-Flag, or plasmids combination pIKKi-Myc and pBFRF1-Flag, then cells lysates were immunoprecipitated with anti-Flag mAb or mouse nonspecific IgG, and results demonstrated that BFRF1 interacted with both the overexpressed (**Figure 5B**) and endogenous IKKi (**Figure 5C**).

To investigate whether BFRF1 also can interact with the signal protein(s) of the RLR signal pathway to inhibit the transcriptional activation of IFN-β, HEK293T cells were co-transfected with BFRF1 and RIG-I, IPS-1, TBK1, TRAF3, or IRF3 expression plasmid, then cell lysates were immunoprecipitated with anti-Flag mAb or mouse nonspecific IgG. As results, BFRF1 could not associate with the adaptor protein of RLR signal pathway mentioned above (**Figures 5D–H**). These results revealed that BFRF1 might affect the IFN-β production *via* only interacting with IKKi.

### BFRF1 Cannot Inhibit the IKKi and IRF3 Interaction but Impede the Kinase Activity of IKKi

**Figure 6:**
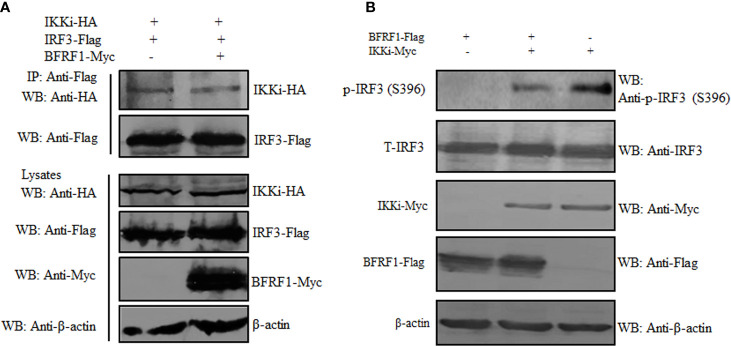
BFRF1 cannot affect the interaction between IKKi and IRF3 but inhibit the kinase activity of IKKi. **(A)** HEK293T cells were co-transfected with plasmids combination of pIKKi-HA/pIRF3-Flag or pIKKi-HA/pIRF3-Flag/pBFRF1-Myc, at 24 h post-transfection, cells were lysed and immunoprecipitated with anti-Flag mAb, then WB analysis was performed using the indicated Abs. **(B)** HEK293T cells was transfected with expression plasmid of BFRF1-Flag or IKKi-Myc, or co-transfected with plasmids combination of pIKKi-Myc/pBFRF1-Flag, at 24 h post-transfection, cells were lysed and WB analysis was performed using the indicated Abs.

The association of BFRF1 with IKKi raised the possibility that BFRF1 may disturb the interaction between IKKi and IRF3. Alternatively, the binding of BFRF1 to IKKi may act as a pseudosubstrate for IKKi to inhibit its kinase activity. To address these possibilities, HEK293T cells were co-transfected with plasmids combination of pIKKi-HA/pIRF3-Flag or pIKKi-HA/pIRF3-Flag/pBFRF1-Myc, then Co-IP assays were carried out, and results found that BFRF1 could not affect IKKi and IRF3 interaction (**Figure 6A**). To further detect whether the BFRF1 and IKKi association influence the kinase activity of IKKi, HEK293T cells were transfected with the expression plasmid of BFRF1-Flag or IKKi-Myc, or co-transfected with plasmids combination pIKKi-Myc/pBFRF1-Flag, and results showed that BFRF1 could inhibit the kinase activity of IKKi, since the IKKi-mediated phosphorylation of IRF3 was restrained (**Figure 6B**). Accordingly, these data disclosed the engagement of BFRF1 could not affect the interaction of IKKi and IRF3 but block the catalytic activity of IKKi.

### BFRF1 Inhibits the Activation of IRF3

**Figure 7:**
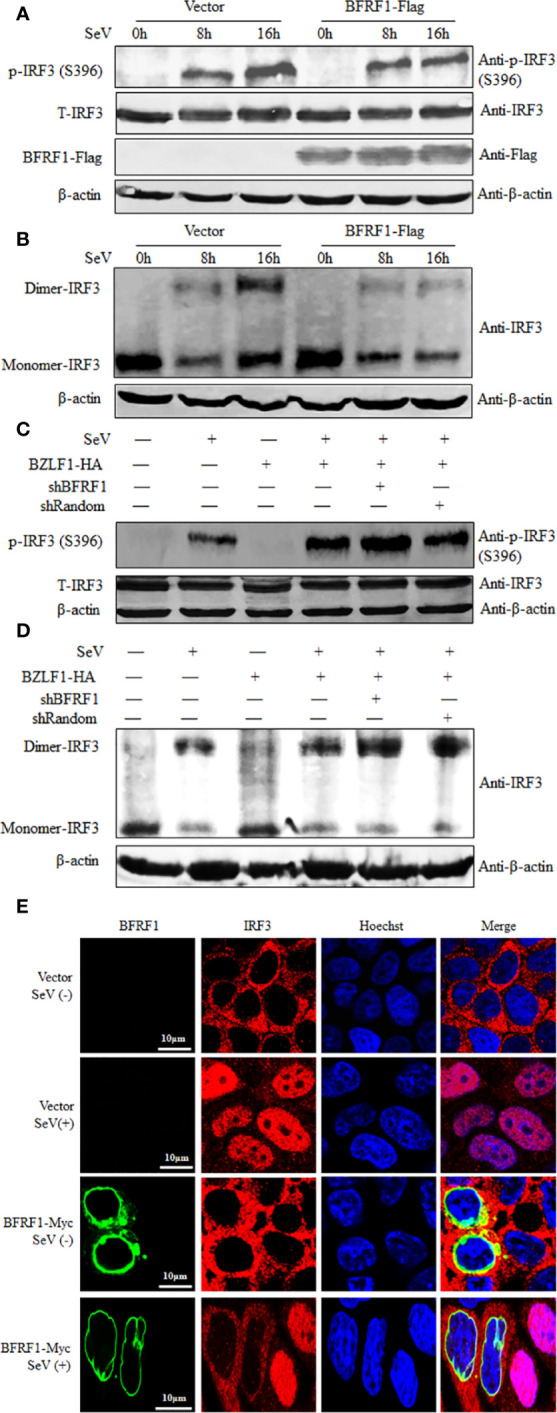
BFRF1 blocks the SeV-induced phosphorylation, dimerization, and nuclear translocation of IRF3. **(A, B)** HEK293T cells were transfected with vector or Flag-tagged BFRF1 expression plasmid. At 24 h post-transfection, cells were mock-infected or infected with 100 HAU/ml SeV for 8 or 16 h. Then, whole-cell extracts were prepared and subjected to IRF3 phosphorylation analysis **(A)** for phosphorylated IRF3 (Ser396), total IRF3, β-actin, BFRF1-Flag, and native PAGE analysis **(B)** for IRF3 dimerization, using related Abs as indicated. **(C, D)** Hone1-EBV cells were co-transfected with BZLF1-HA expression plasmid and pSuper-shBFRF1-retro-puro or pSuper-shRandom-retro-puro expression plasmid. At 24 h post-transfection, cells were mock-infected or infected with 100 HAU/ml SeV for 16 h. Then, whole-cell extracts were prepared and subjected to IRF3 phosphorylation analysis **(C)** and native PAGE analysis **(D)**, as indicated in **(A, B)**, respectively. **(E)** HeLa cells were transfected with vector or Myc-tagged BFRF1 expression plasmid, at 24 h post-transfection, cells were mock-infected or infected with 100 HAU/ml SeV for 16 h. Cells were then probed with primary Abs mouse anti-Myc mAb and rabbit anti-IRF3 pAb, and secondary Abs FITC-conjugated goat anti-mouse IgG (green) and Cy5-conjugated goat anti-rabbit IgG (red), respectively. Cells were counterstained with Hoechst to visualize the nuclear DNA (blue). Images were obtained by confocal microscopy using a 63× lens objective. All scale bars indicate 10 μm. Statistical analysis of the subcellular localization of IRF3 in the absence or presence of BFRF1 was shown in **Table 1**.

Generally, IRF3 Ser396 is targeted for phosphorylation following virus infection, which plays an essential role in IRF3 activation (74, 75). Therefore, to dissect whether IRF3 phosphorylation is inhibited by BFRF1, HEK293T cells were transfected with BFRF1-Flag expression plasmid or vector, then cells were mock-treated or treated with 100 HAU/ml SeV for 8 or 16 h, and cells were collected for WB analysis using phospho-IRF3 (Ser396) Ab. As results, SeV infection for 8 or 16 h could induce the accumulation of IRF3 Ser396, while this phosphorylation was significantly inhibited by BFRF1 when SeV infection for 16 h (**Figure 7A**). Since IRF3 dimer formation is a consequence of IRF3 phosphorylation, we continued to test whether BFRF1 could inhibit SeV-induced IRF3 dimerization, and result showed that IRF3 dimerization was also obviously reduced by the expression of BFRF1 when cells were treated with SeV for 16 h (**Figure 7B**). To continue verify the BFRF1 inhibitory effect of IRF3 phosphorylation and dimerization during EBV lytic infection, Hone1-EBV cells were co-transfected with the BZLF1 expression plasmid and pSuper-shBFRF1-retro-puro or pSuper-shRandom-retro-puro expression plasmid. Compared with lane 4 of **Figures 7C, D**, the phosphorylation of IRF3 Ser396 (**Figure 7C**, lane 5) and dimerization of IRF3 (**Figure 7D**, lane 5) could be up-regulated by knocking down the expression of BFRF1 after inducing EBV into lytic infection in Hone1 cells, verifying the experimental results at the transfection level of **Figures 7A, B**. These results demonstrated that BFRF1 could effectively prevent the phosphorylation and dimerization of IRF3 during EBV lytic infection, which was also consistent with the results of **Figures 2A, B**, again confirming EBV lytic infection could inhibit the production of IFN-β and downstream ISG, and BFRF1 played a certain important role in this process.

Upon phosphorylation, IRF3 dimerizes and translocates into the nucleus, where it can form a complex with CBP/p300 and act as a transcriptional factor. The holocomplex then binds to IRF3 sites in the IFN-β promoter, and eventually activates the transcription of IFN-β (76). As the aforementioned results indicated that BFRF1 repressed the phosphorylation and dimerization of IRF3, we subsequently investigated whether BFRF1 inhibited SeV-induced IRF3 nuclear translocation. HeLa cells were transfected with BFRF1-Myc expression plasmid or vector, then cells were mock-treated or treated with 100 HAU/ml SeV for 16 h, and IFA was performed using confocal microscope. As shown in **Figure 7E** and statistical analysis of the subcellular localization in **Table 1** that is widely applied in many studies (70, 77–80), IRF3 was localized exclusively in the cytoplasm in mock-infected HeLa cells, while most of IRF3 translocated into the nucleus after SeV stimulation. However, IRF3 was restricted in the cytoplasm in BFRF1 expressing cells. These results indicated that SeV-induced IRF3 nuclear translocation was conspicuously inhibited by BFRF1.

**Table 1:** Subcellular localization of IRF3 in the presence of EBV BFRF1.

Host protein	Cells transfected with vector or BFRF1 expression plasmid	Cells treated with SeV	Total number of cells transfected with vector or BFRF1 expression plasmid	Subcellular localization pattern of IRF3 in cells transfected with vector or BFRF1 expression plasmid	Number of subcellular localization change of IRF3 in cells transfected with vector or BFRF1 expression plasmid	Percentage of subcellular localization change of IRF3 in cells transfected with vector or BFRF1 expression plasmid
IRF3	Vector	−	20	Pan-cytoplasmic	0	0
IRF3	Vector	+	20	Nuclear	1	5
IRF3	BFRF1-Myc	−	20	Pan-cytoplasmic	0	0
IRF3	BFRF1-Myc	+	20	Pan-cytoplasmic or pan-cellular	15	75

HeLa cells were transfected with Myc vector or BFRF1-Myc expression plasmid. At 24 h post-transfection, cells were treated with or without 100 HAU/ml SeV for 16 h. Then, cells were examined for the subcellular localization pattern of IRF3 by IFA.

## Discussion

Innate immunity is an ancient and conserved defense that rapidly responds to pathogen invasion. However, viruses have evolved diverse strategies to overcome the host antiviral responses for their survivals. EBV is the first identified human cancer virus, which has developed a series of elaborate and sophisticated strategies to escape host immune system (12, 81–85). In addition to the EBV-encoded products (BGLF4, BZLF1, BRLF1, LMP2A, LMP2B, and miR-BART6-3p) described in the introduction, EBV-encoded BILF4 (LF2) also can bind to IRF7 to restrain its activity and subsequent IFN promoter activation (86), EBNA2 can inhibit IFN-I signaling by reducing or abolishing the expression of distinct ISGs (87). Besides, EBV-induced host miR-146 can target TRAF6, IRAK1, and IRAK2 to attenuate IFN-I production in macrophages (88).

**Figure 8:**
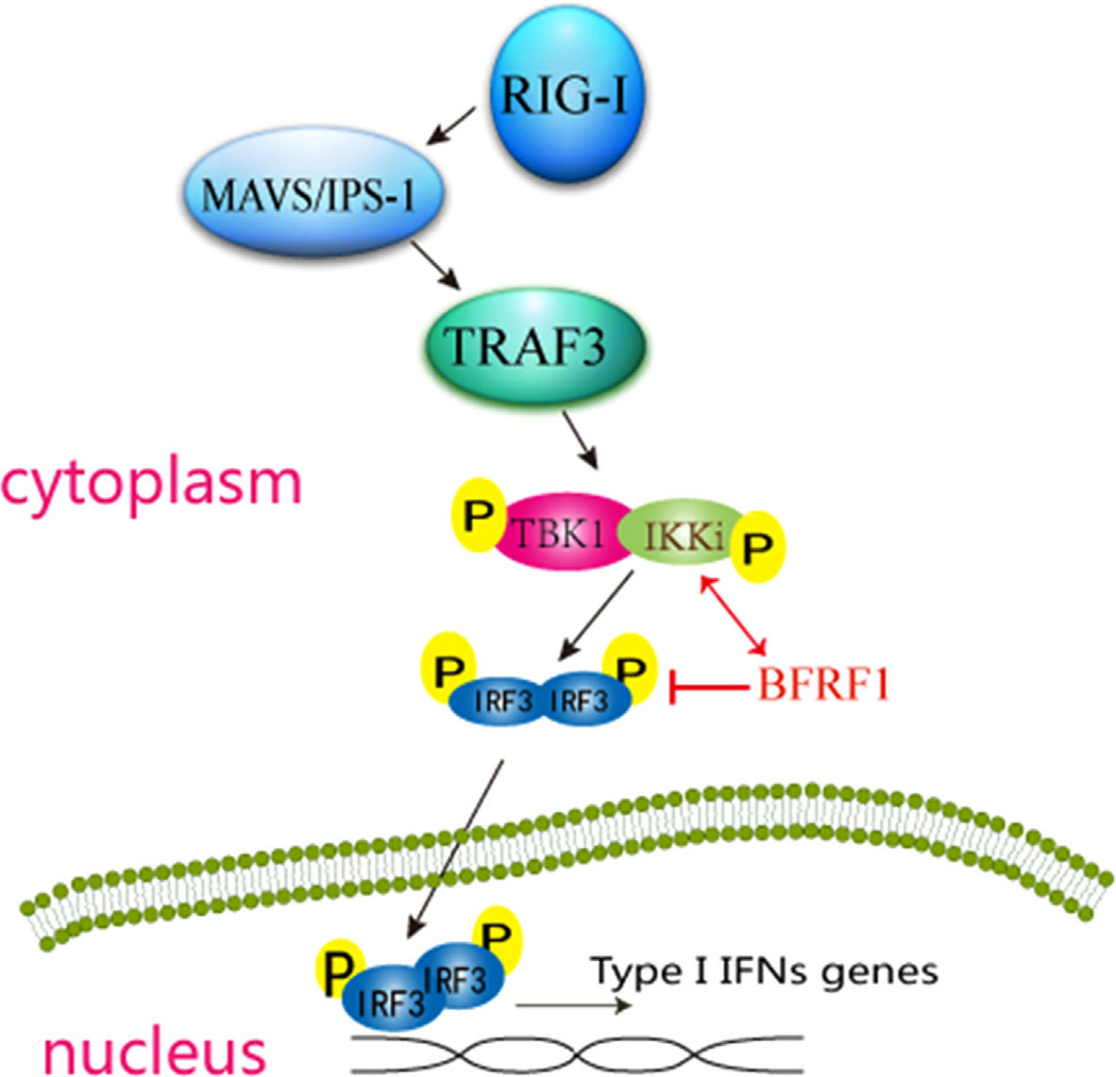
Schematic diagram of EBV early protein BFRF1 inhibiting RLR-mediated IFN-β signaling pathway. RNA helicase RIG-I is activated by upstream stimulation, which signals through IPS-1 to activate the kinases TBK1 and IKKi, then TBK1/IKKi complex phosphorylates IRF3, this leads to the induction of phosphorylation, dimerization, and the nuclear translocation of IRF3 and subsequent IFN-I production. In this study, EBV-encoded early lytic protein BFRF1 is shown to repress IFN-β transcriptional activity by interacting with IKKi to inhibit the phosphorylation, dimerization, and nuclear accumulation of IRF3. The red line (↕) shows that BFRF1 interacts with IKKi, and red line (T) shows that BFRF1 suppresses the phosphorylation, dimerization, and nuclear translocation of IRF3.

To further explore whether there are other EBV-encoded proteins can inhibit RLR-mediated IFN-β production, a screening of EBV proteins for their abilities to block SeV-induced activation of IFN-β promoter was performed. Interestingly, EBV early lytic protein BFRF1 was found to significantly inhibit SeV-stimulated IFN-β production. While previous study demonstrates that BFRF1 is essential for the efficient primary viral envelopment and egress (28), it’s not known that BFRF1 is involved in the regulation of IFN-I signaling pathway. In the present study, we showed that BFRF1 blocked the activation of IRF3 promoter (but not IRF7 or NF-κB) through specifically targeted the IKK-related kinase IKKi (but not TBK1) and affected its kinase activity but did not alter the interaction of IKKi and IRF3, which finally inhibited RLR-induced phosphorylation, dimerization, and nuclear translocation of IRF3. These results are similar with previous reports that viral proteins can inhibit the RLR pathway at the level of IRF3 upstream but cannot restrain the downstream adaptor mediated-promoter activity (89–91). Taken together (as shown in **Figure 8**), these data indicated that BFRF1 abrogated IFN-β production by blocking IRF3 activation, which may be important for viral maturation and nuclear egress.

The result that BFRF1 targets to IKKi but not TBK1 was unpredictable, because TBK1 takes a predominant role in the production of IFN-I in response to dsRNA and viral stimulation (92–94) However, the IFN-I production was not influenced in TBK1-deficient macrophages (93), suggesting IKKi is also essential for the optimal induction of IFN-I. Therefore, the roles of TBK1 and IKKi indicate that IRF3 phosphorylation in cells involves a complicated requiring of both kinases (95). Here, we speculate that BFRF1 may bind to the kinase domain of IKKi and intercept its autocatalytic activity to phosphorylate IRF3, or BFRF1 may sequester IKKi into an inactive complex (95, 96). The interaction of BFRF1 with IKKi may also compete for the association of IKKi to IRF3 (but not IRF7) to inhibit IRF3 (but not IRF7) binding to its corresponding binding site on the IFN-β promoter (97). It is conceivable that BFRF1 interacting with IKKi may also disturb the function of TBK1. After further investigation (**Figure 6**), we confirmed that the association of BFRF1 with IKKi did not disturb the binding of IKKi to IRF3, but BFRF1 could block the catalytic activity of IKKi. Accordingly, the specificity of BFRF1 for IKKi would be favorable for persistent EBV to circumvent the host innate immunity.

IRF3 is an essential transcriptional factor in antiviral process, which is required for the expression of IFN-I and many other genes. Previous works have suggested a dual phosphorylation-dependent mechanism regulates the activation of IRF3. Specifically, Ser 385 and 386 comprise “site 1,” and Ser 396, 398, 402, 405, and Thr 404 comprise “site 2” (94, 98). The phosphorylation of both site 1 and site 2 are required for the full activation of IRF3 (99). Upon viral infection, cellular TBK1- and IKKi-mediated phosphorylation of serines 385 and 386 and the serine/threonine cluster between amino acids 396 and 405 of IRF3 lead to its conformational change and activation (100–102). Here, the Ser 396 is essential for IRF3 activation, especially after viral infection. Therefore, we investigated whether the phosphorylation of IRF3 (Ser 396) was inhibited by BFRF1 during ectopic expression and EBV lytic infection, and our results confirmed this speculation. Of course, it does not exclude the possibility that the IRF3 phosphorylation of other sites are also inhibited.

Since IRF3 plays a central role in the innate immune response, it’s not surprising many viral proteins can disrupt IRF3 activation. HSV-1 VP24 protein binds IRF3 to prevent TBK1/IRF3 interaction and block the phosphorylation and dimerization of IRF3 during viral infection (103). Encephalomyocarditis virus 3C protein disrupts the TANK-TBK1-IKKi-IRF3 tetramer formation and decreases TBK1- and IKKi-mediated IRF3 phosphorylation and IFN-I production (104). Heartland virus NSs protein interacts with TBK1 and blocks TBK1/IRF3 interaction to constrain the activation of IRF3 (105). Paramyxovirus V protein interacts with IKKi/TBK1 to act as their substrates to inhibit IRF3 phosphorylation and its activation (106). Thrombocytopenia syndrome bunyavirus nonstructural protein NSs can interact with TBK1 to sequester the IKK complex to restrict phosphorylated IRF3 translocates into the nucleus (107). While in this study, BFRF1 was proved to interact with IKKi, but may not RIG-I, IPS-1, TBK1, TRAF3, or IRF3 (since we cannot completely rule out that the antibodies used in this study may not work well in the Co-IP experiments), to abrogate IFN-β production by blocking IRF3 phosphorylation, dimerization, and nuclear translocation.

It’s believed that analyzing the transcriptional expression level of BFRF1 in plasmid transfected cells and EBV lytic infected cells induced from EBV latent cells can help us to confirm whether the plasmid transfected BFRF1 has similar inhibitory effect of IFN-β production with that of BFRF1 during EBV infection. Although we did not have EBV-positive Akata cell line (EBV latent B cells), EBV-positive Hone1 cell line was selected as a representative cellular model because it grow better and is suitable for related *in vitro* and *in vivo* experiments (108), which is also widely used in the related studies of EBV lytic infection from latency (109–115). Simultaneously, studies have shown that the expression levels of BFRF1 at different time points in plasmid transfected cells are consistent with that of the EBV lytic infected cells induced from EBV latent cells (116, 117). Therefore, it can be concluded that the inhibitory effect of BFRF1 on IFN-β production during plasmid transfection is not an illusion, and BFRF1 also has similar inhibitory effect on IFN-β during EBV lytic infection, which was confirmed in EBV-positive Hone1 cells of our study (**Figures 2**, **7**).

As mentioned above, BFRF1 is reported to take a very important role in the nucleocapsid release and viral maturation of EBV, and deletion of BFRF1 will lead to a serious decrease in viral production (27, 28). In addition, our study found that overexpression of BFRF1 could down-regulate IFN-β production, and knocking down the expression of BFRF1 in EBV lytic infected Hone1 cells could increase IFN-β production, which is speculated inevitably inhibit the proliferation of EBV. Therefore, if the cells are infected with wild-type EBV and BFRF1 knocked-out EBV, or BFRF1 is knocked down in EBV lytic infection cells induced from EBV latent Hone1 cells, it cannot directly make a conclusion that whether the decrease of EBV production is directly caused by the BFRF1 inhibitory effect of IFN-β, because BFRF1 knockout or knockdown itself will limit the release and maturation of EBV nucleocapsid, which finally reduces the viral production.

To date, six EBV-encoded proteins (BZLF1, BGLF4, LMP2A, LMP2B, LF2, and BRLF1) and three EBV-related RNAs (EBNA2, miR-146, and miR-BART-3p) have been implicated in inhibiting the production of IFN-I. In this study, the EBV early lytic protein BFRF1 was demonstrated to be a novel antagonist of IFN-β production, with evidence that BFRF1 regulated the interferon antiviral response by inhibiting IRF3 activation. This finding will lead to a better understanding of the mechanisms employed by EBV to dampen host antiviral signaling and provide information for the development of therapeutic interventions to modulate EBV pathogenesis.

## Data Availability Statement

The raw data supporting the conclusions of this article will be made available by the authors, without undue reservation, to any qualified researcher.

## Author Contributions

MSC and MLL conceived and designed the experiments. PW, YXD, YJG, ZX, YWL, XWO, LX, MJL, JYZ, BLL, LH, and SYD performed the experiments. MLL, MSC, and PW analyzed the data. TP consulted and advised on the research. PW, MSC, and MLL wrote the paper. All authors contributed to the article and approved the submitted version.

## Funding

This work was supported by grants from the National Natural Science Foundation of China (81772179); the Natural Science Foundation of Guangdong Province (2114050001144, 2019A1515010395 and 2018A0303130257); the Regular University Distinguished Innovation Project from Education Department of Guangdong Province, China (2018KTSCX184 and 2020KTSCX098); the Scientific Research Project of Shenzhen Health and Family Planning System (SZBC2018009); the Guangzhou Health & Medical Collaborative Innovation Program (201803040007); the Guangzhou Innovation & Entrepreneurship Leading Team Program (CYLJTD-201602); the Guangzhou Entrepreneurship Leading Talents Program (LYC201315); the Science and Technology Program of Guangzhou Development District (2018-L081); the High-Level Universities Academic Backbone Development Program of Guangzhou Medical University; and the National, Provincial and College Training Programs of Innovation and Entrepreneur-ship for Undergraduates in Guangzhou Medical University (2018A113, 2019A096, 2019A107, and S201910570088).

## Conflict of Interest

TP was employed by South China Vaccine Corporation Limited.

The remaining authors declare that the research was conducted in the absence of any commercial or financial relationships that could be construed as a potential conflict of interest.

## Supplementary Material

Supplementary Figure 1 Original western blot results of Fig. 1.

Supplementary Figure 2 Original western blot results of Fig. 3.

Supplementary Figure 3 Original western blot results of Fig. 4A-B.

Supplementary Figure 4 Original western blot results of Fig. 4C-D.

Supplementary Figure 5 Original western blot results of Fig. 4E-F.

Supplementary Figure 6 Original western blot results of Fig. 5B-C.

Supplementary Figure 7 Original western blot results of Fig. 5D-H.

Supplementary Figure 8 Original western blot results of Fig. 6A.

Supplementary Figure 9 Original western blot results of Fig. 6B.

Supplementary Figure 10 Original western blot results of Fig. 7A-B.

Supplementary Figure 11 Original western blot results of Fig. 7C-D.
