# Supplementary figures and images for: Epstein-Barr Virus Early Protein BFRF1 Suppresses IFN-β Activity by Inhibiting the Activation of IRF3
Source: Front Immunol. 2020 Dec 17;11:513383. doi: 10.3389/fimmu.2020.513383 (PMC7774019; doi:10.3389/fimmu.2020.513383)

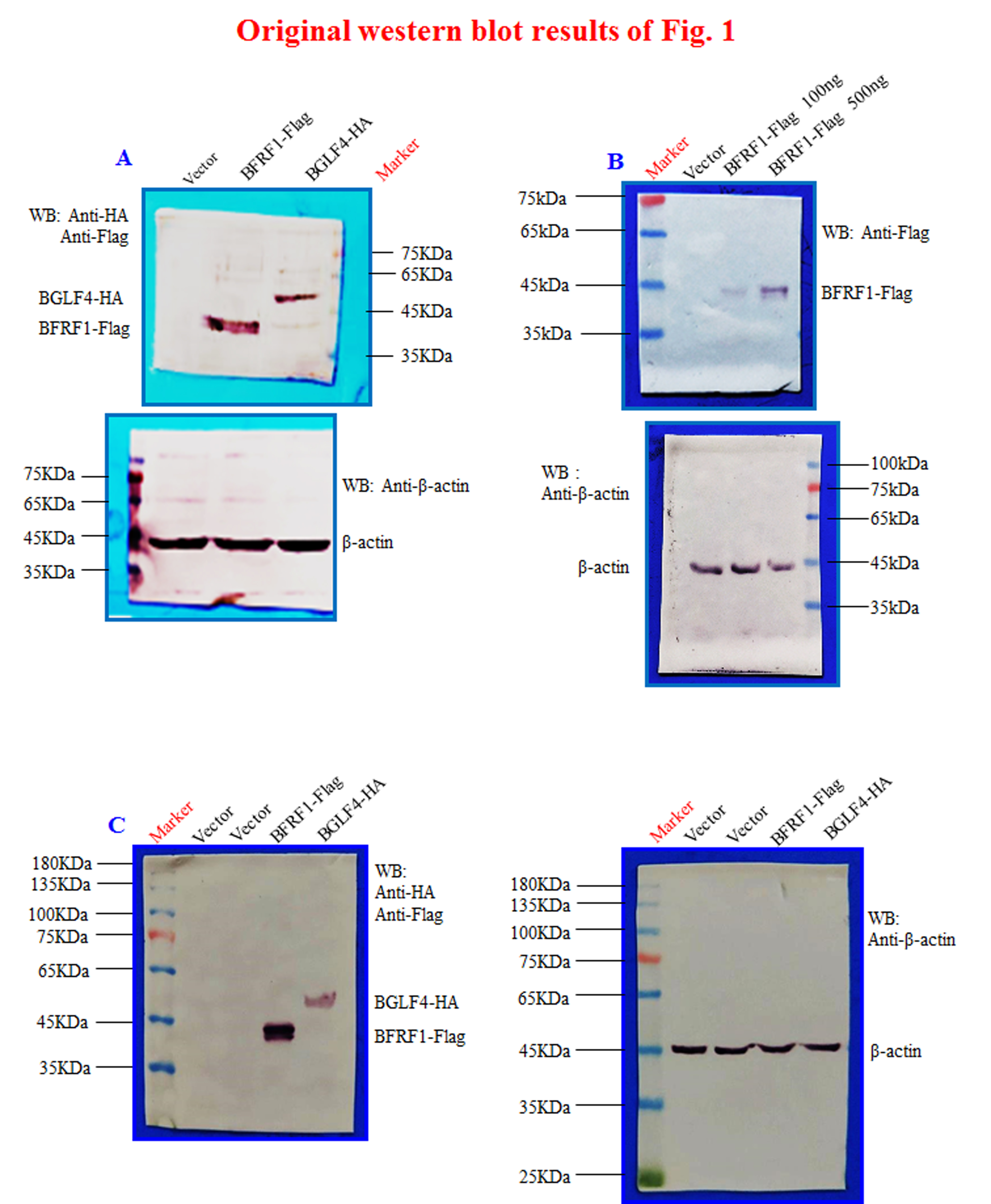

Supplement: Supplementary Figure 1 — Original western blot results of Fig. 1. [file Image_1.jpeg]

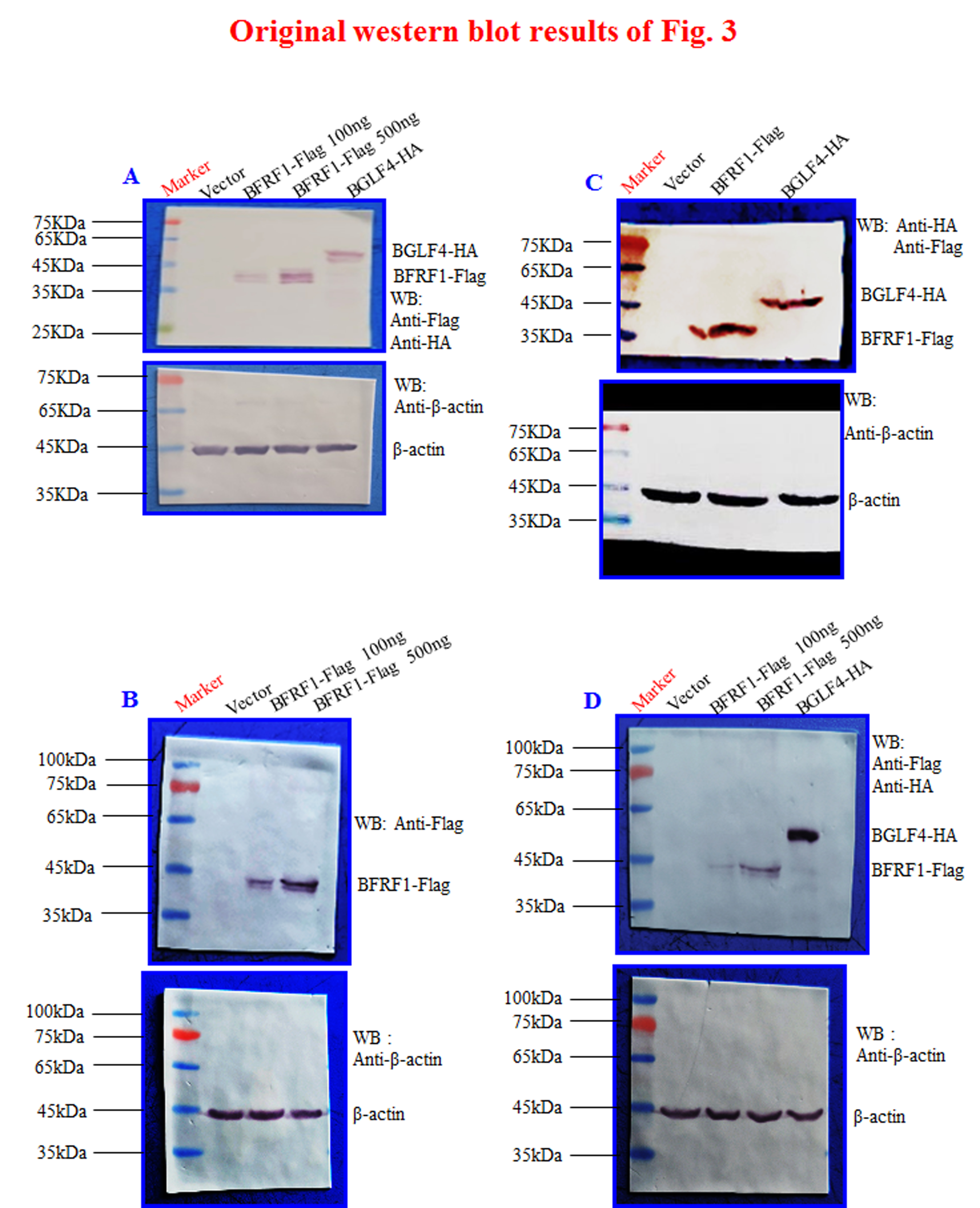

Supplement: Supplementary Figure 2 — Original western blot results of Fig. 3. [file Image_2.jpeg]

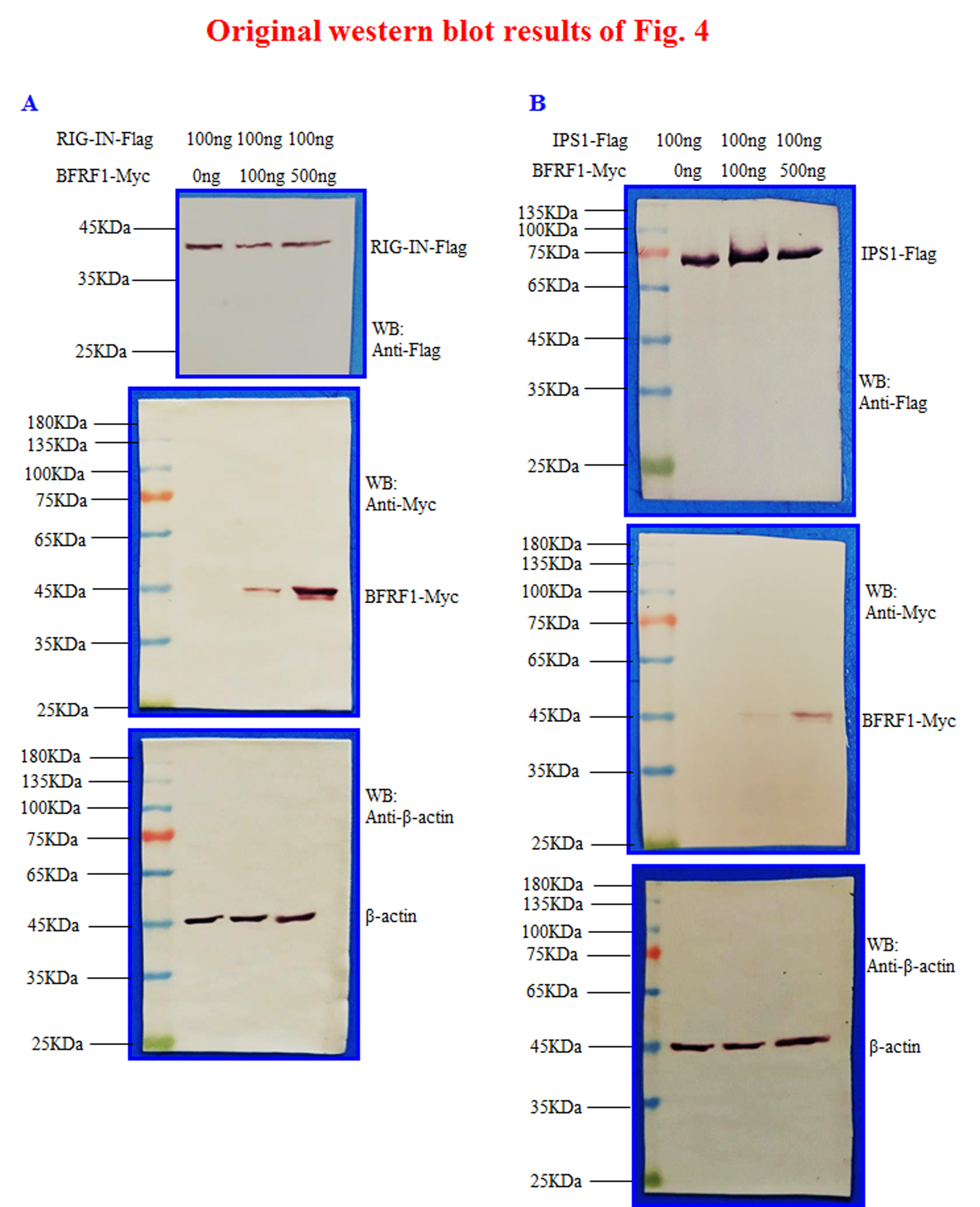

Supplement: Supplementary Figure 3 — Original western blot results of Fig. 4A-B. [file Image_3.jpeg]

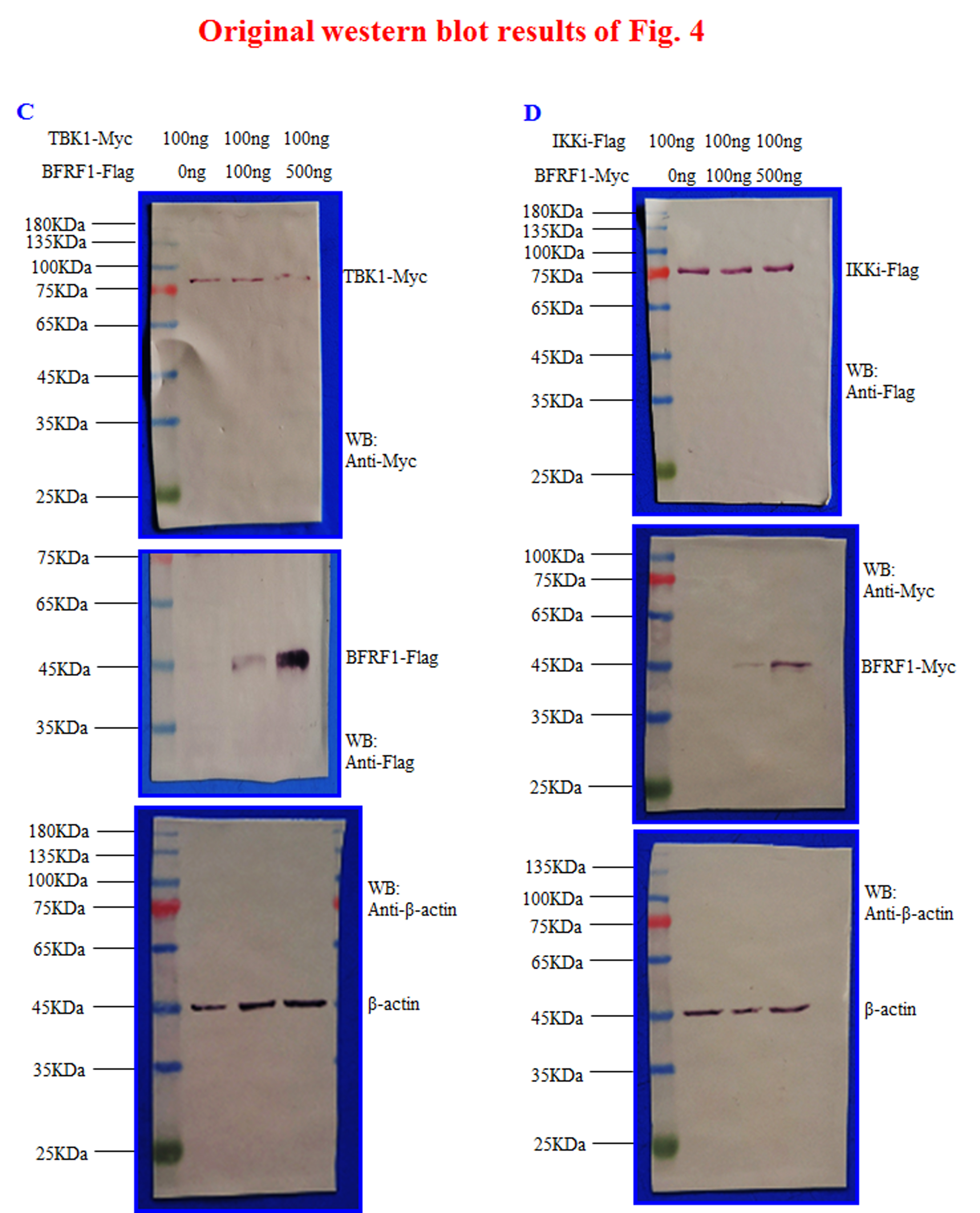

Supplement: Supplementary Figure 4 — Original western blot results of Fig. 4C-D. [file Image_4.jpeg]

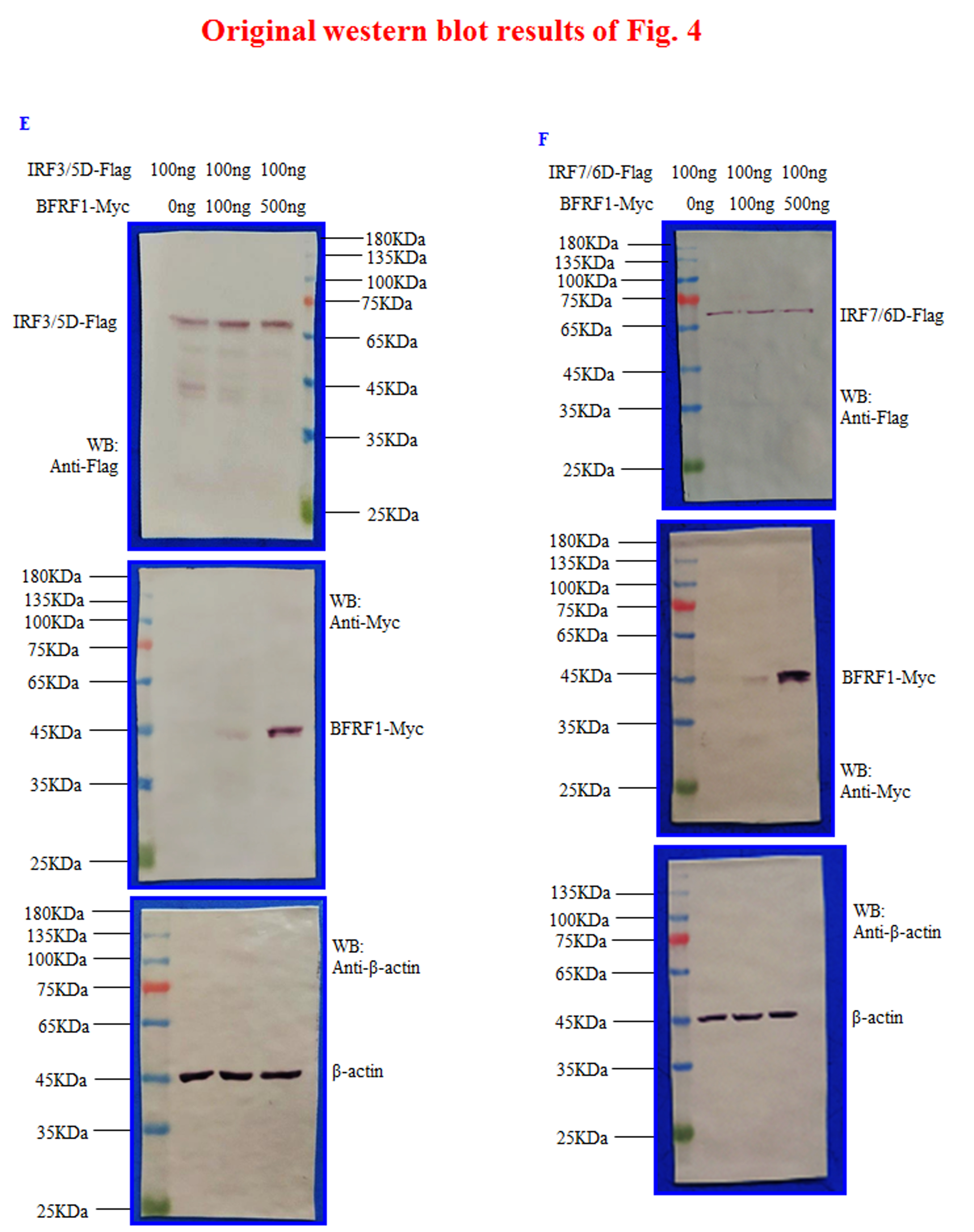

Supplement: Supplementary Figure 5 — Original western blot results of Fig. 4E-F. [file Image_5.jpeg]

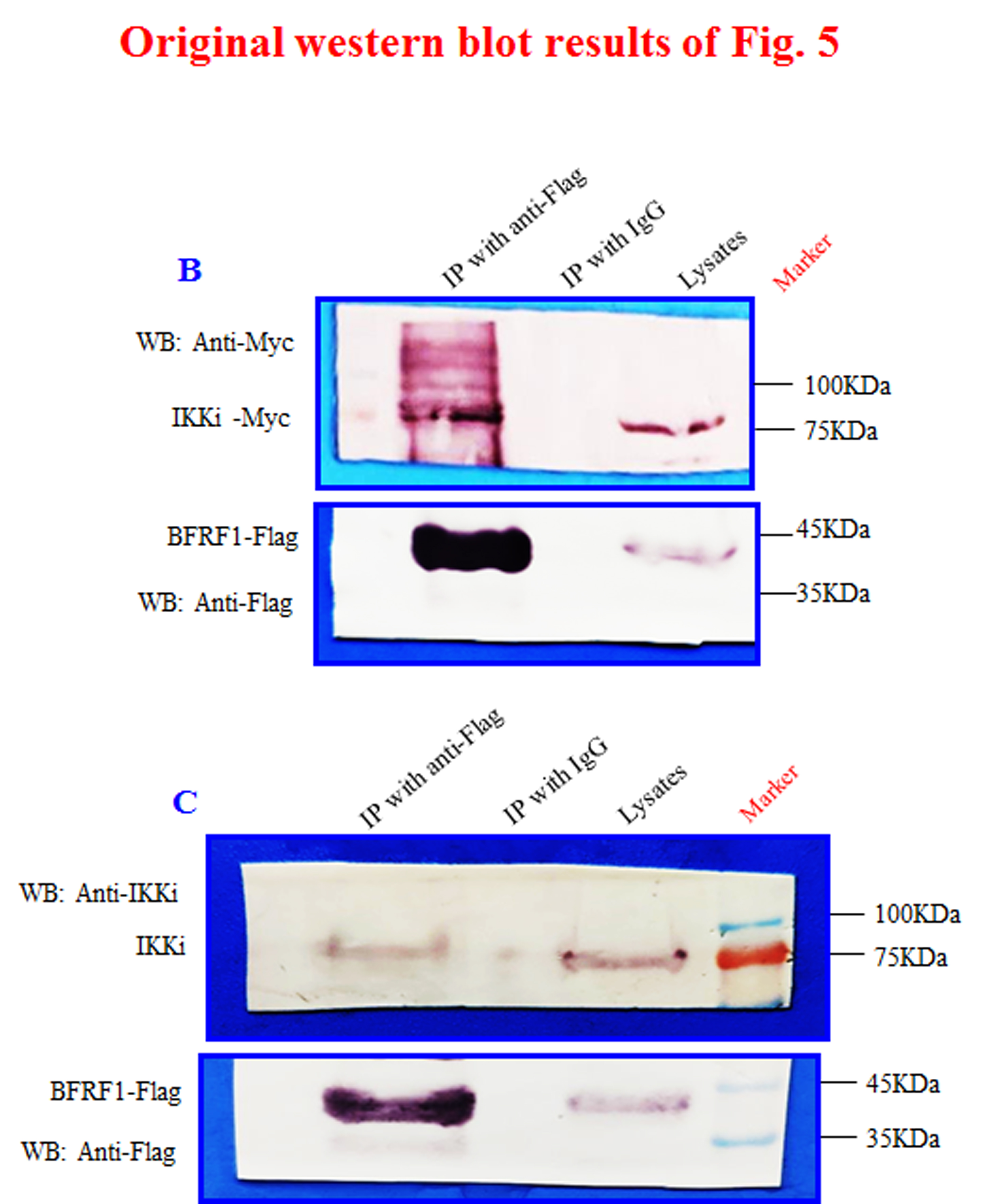

Supplement: Supplementary Figure 6 — Original western blot results of Fig. 5B-C. [file Image_6.jpeg]

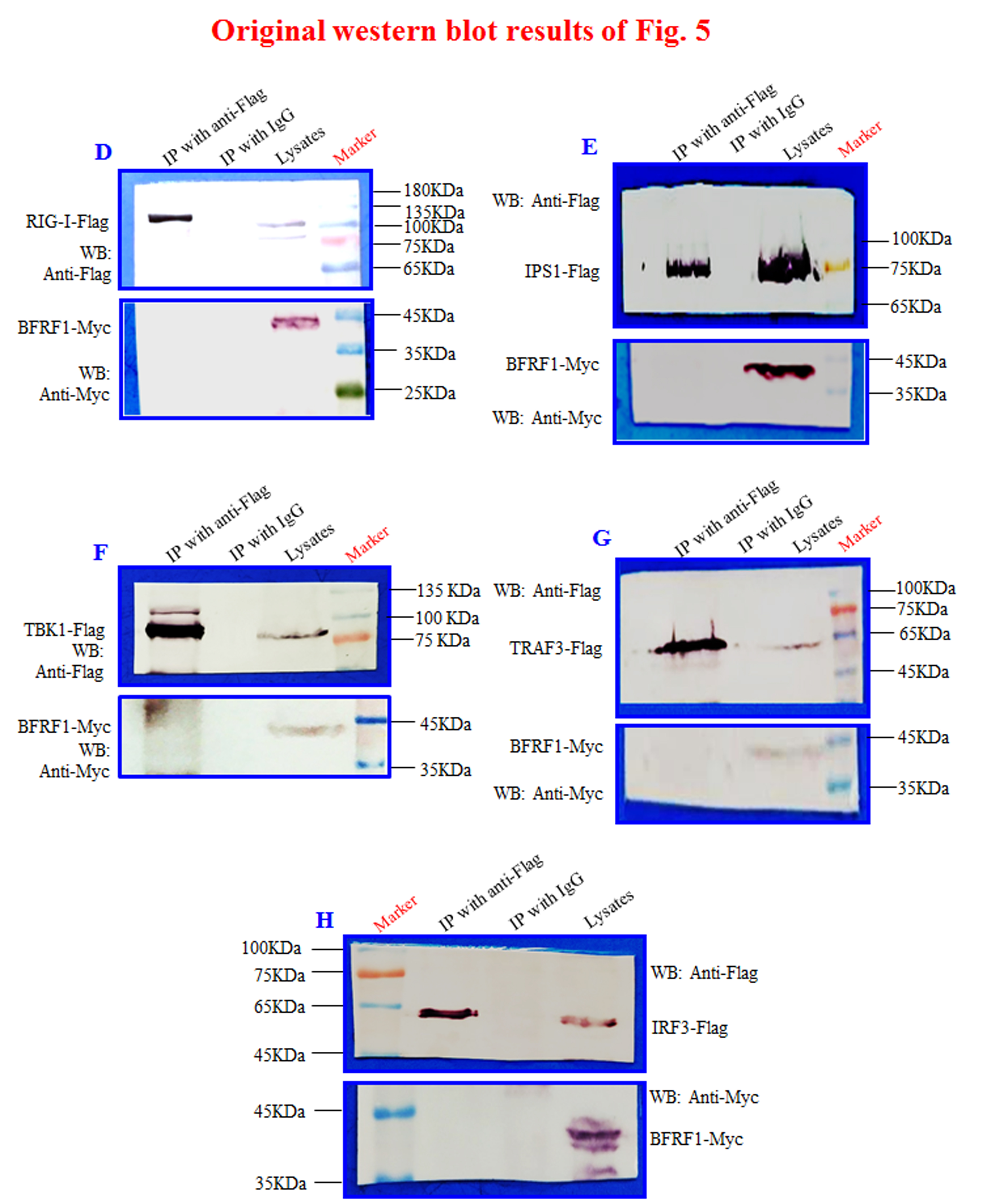

Supplement: Supplementary Figure 7 — Original western blot results of Fig. 5D-H. [file Image_7.jpeg]

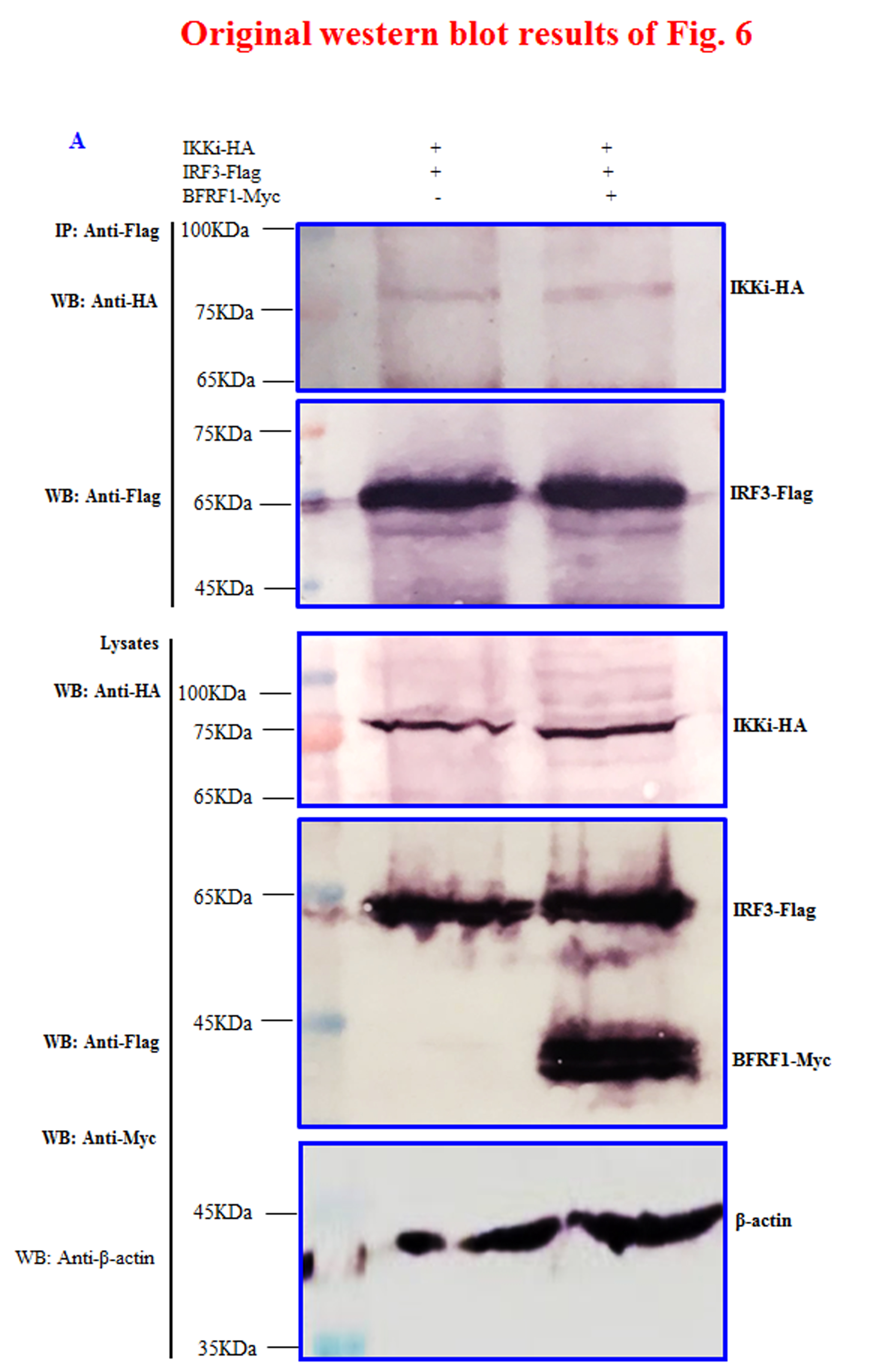

Supplement: Supplementary Figure 8 — Original western blot results of Fig. 6A. [file Image_8.jpg]

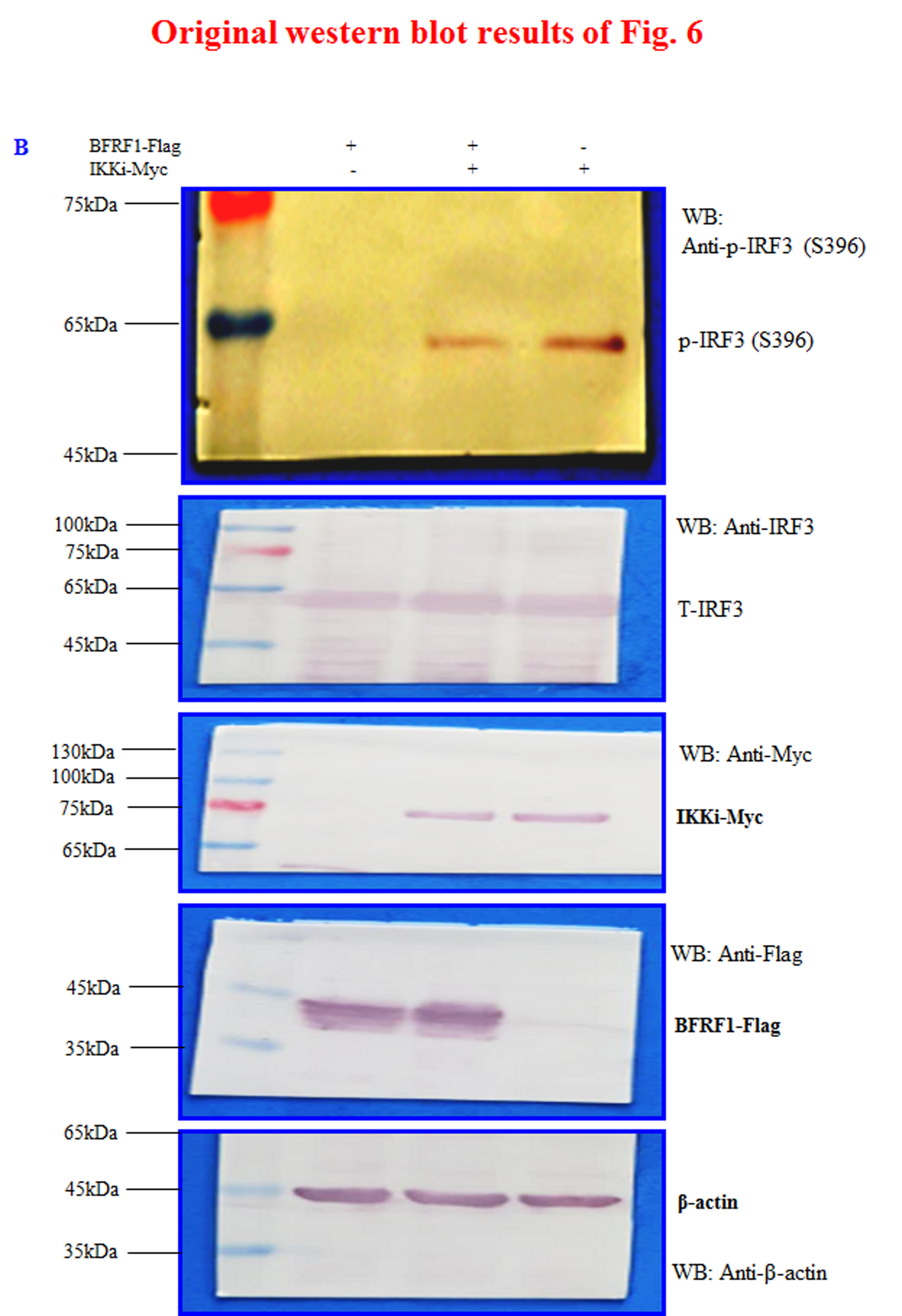

Supplement: Supplementary Figure 9 — Original western blot results of Fig. 6B. [file Image_9.jpeg]

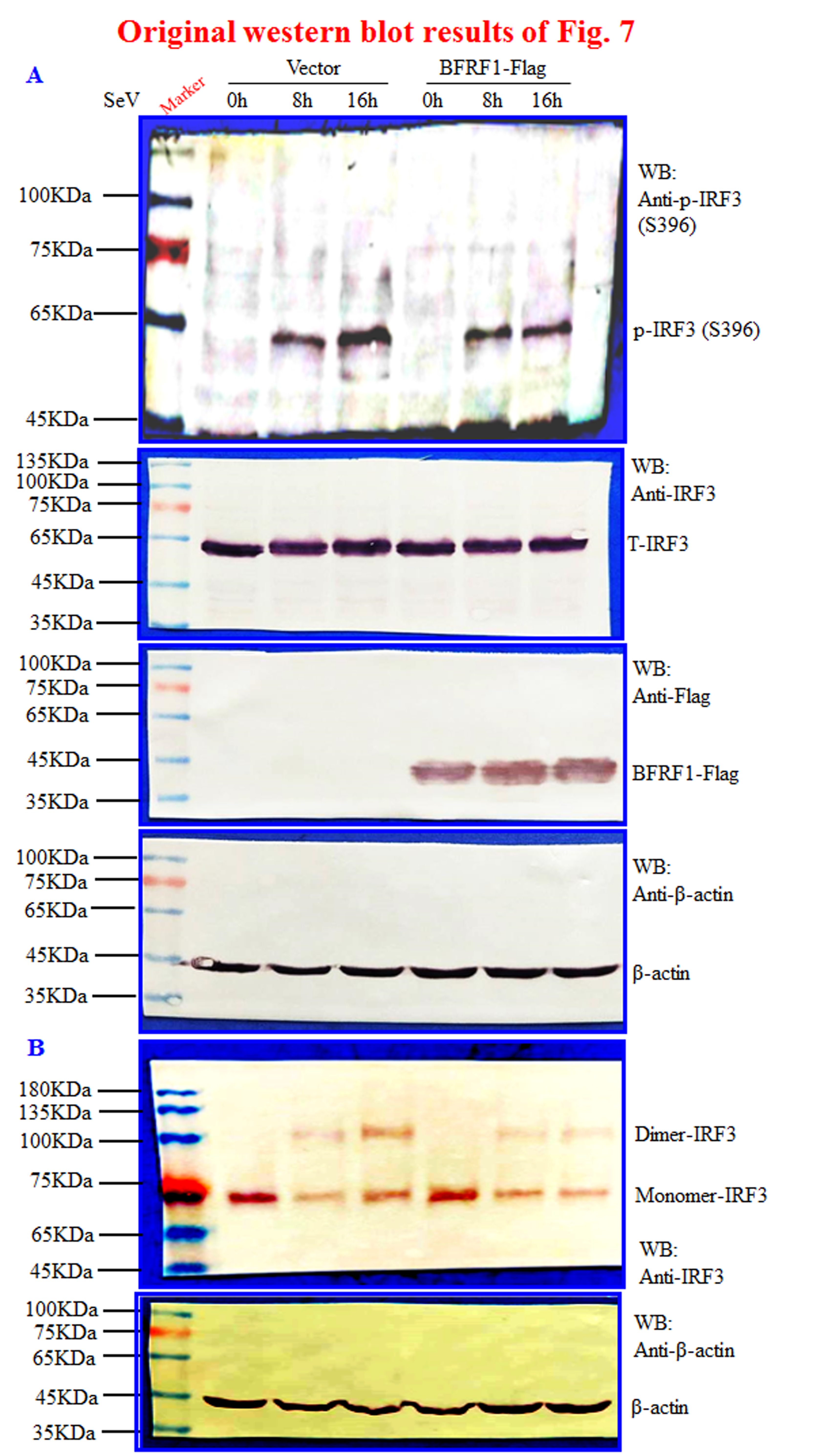

Supplement: Supplementary Figure 10 — Original western blot results of Fig. 7A-B. [file Image_10.jpeg]

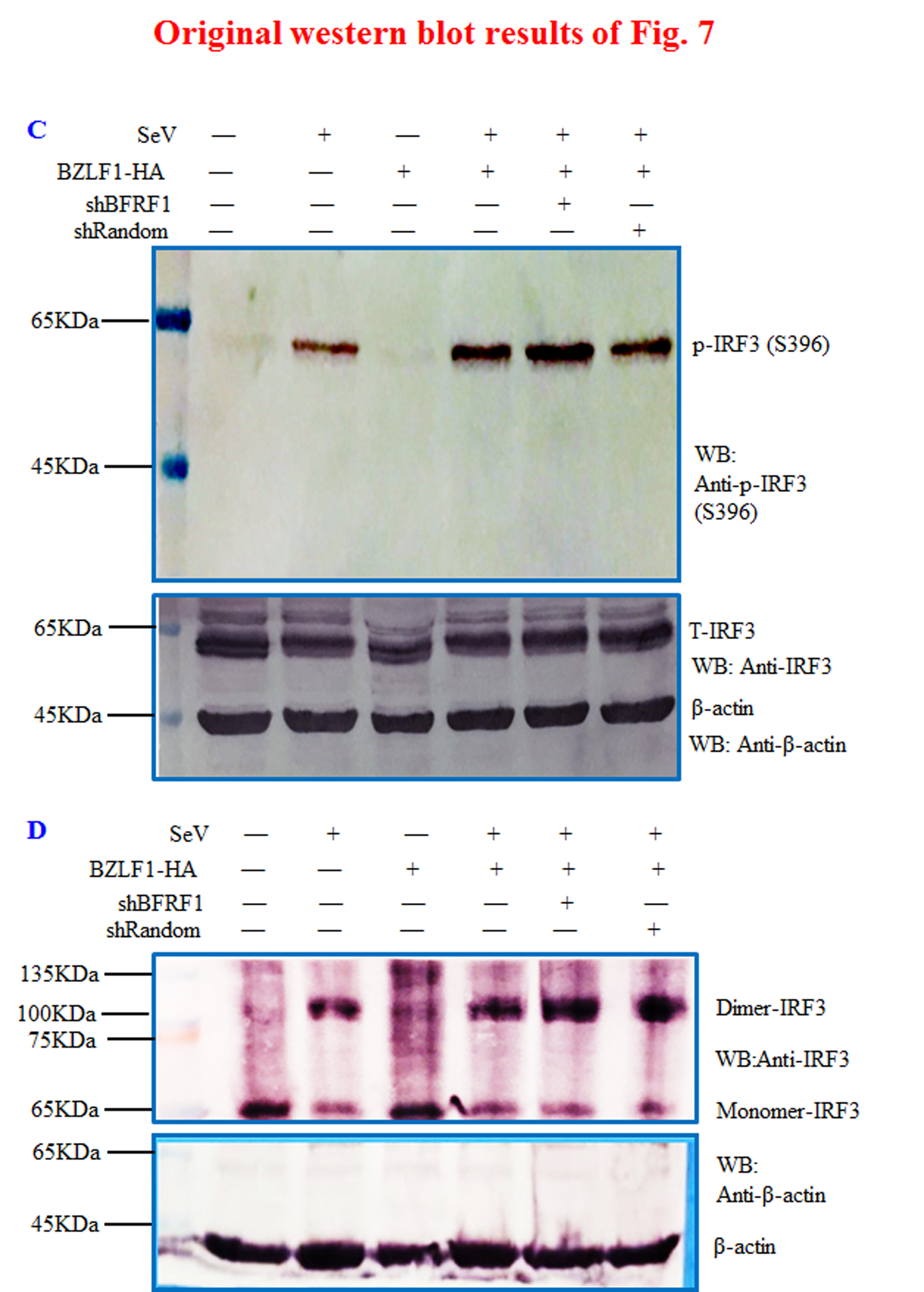

Supplement: Supplementary Figure 11 — Original western blot results of Fig. 7C-D. [file Image_11.jpeg]
